# Synthesis, Biological and In Silico Studies of a Tripodal Schiff Base Derived from 2,4,6-Triamino-1,3,5-triazine and Its Trinuclear Dy(III), Er(III), and Gd(III) Salen Capped Complexes

**DOI:** 10.3390/molecules26144379

**Published:** 2021-07-20

**Authors:** Uchechukwu Susan Oruma, Pius Oziri Ukoha, Chiamaka Peace Uzoewulu, Joseph Chinedum Ndefo, Sabastine Chinweike Ugwuoke, Nkechinyere Nwanneka Ukwueze, Tochukwu Emmanuella Eze, Lilian Chinenye Ekowo, Florence Uchenna Eze, Uchenna Vivian Chinaegbomkpa, Sunday Nwankwo Okafor, Chigozie Julius Ezeorah

**Affiliations:** 1 Coordination Chemistry and Inorganic Pharmaceuticals Unit, Department of Pure and Industrial Chemistry, University of Nigeria, Nsukka 410001, Nigeria; susan.oruma@unn.edu.ng (U.S.O.); pius.ukoha@unn.edu.ng (P.O.U.); 2 Department of Pure and Industrial Chemistry, University of Nigeria, Nsukka 410001, Nigeria; chiamaka.uzoewulu@unn.edu.ng (C.P.U.); nkechinyere.ukwueze@unn.edu.ng (N.N.U.); tochyodike@gmail.com (T.E.E.); chinenye.ekowo@unn.edu.ng (L.C.E.); florence.ali@unn.edu.ng (F.U.E.); uchenna.chinaegbomkpa@futo.edu.ng (U.V.C.); 3 Department of Science Laboratory Sciences, University of Nigeria, Nsukka 410001, Nigeria; joseph.ndefo@unn.edu.ng; 4 Department of Biochemistry, University of Nigeria, Nsukka 410001, Nigeria; sabastine.ugwoke.88417@unn.edu.ng; 5 Department of Chemistry, Federal University of Technology, Owerri 460114, Nigeria; 6 Department of Pharmaceutical and Medicinal Chemistry, University of Nigeria, Nsukka 410001, Nigeria

**Keywords:** tripodal schiff base, trinuclear Ln(III) complexes, antimicrobial activity, antimalarial activity, docking

## Abstract

A tripodal Schiff base ligand, 2,4,6-Tris(4-carboxybenzimino)-1,3,5-triazine (MT) and its trinuclear Dy(III), Er(III), and Gd(III) complexes were synthesized. These were characterized using UV-visible, IR, ^1^H, and ^13^C NMR spectroscopies, elemental analysis, and molar conductivity measurements. The spectral studies indicate that the ligand is hexadentate and coordinates to the Ln(III) ions through the oxygen atoms of the carboxylic group. The trinuclear complexes were characterized as being bridged by carboxylate anions to the Dy(III), Er(III), and Gd(III) salen centers and displaying a coordination number of six. Biological studies revealed that MT is more active against the test micro-organisms relative to the trinuclear complexes. Acute toxicity studies revealed that MT is safe and has a wide range of effective doses (ED_50_). In vivo antimalarial studies indicate that MT could serve as an effective antimalarial agent since it has parasitemia inhibition of 84.02% at 50 mg/kg and 65.81% at 25 mg/kg, close to the value (87.22%) of the standard drug—Artesunate. Molecular docking simulation studies on the compounds against SARS-CoV-2 (6Y84) and *E. coli* DNA gyrase (5MMN) revealed effective binding interactions through multiple bonding modes. The binding energy calculated for Er(III)MT-6Y84 and Er(III)MT-5MMN complexes showed active molecules with the ability to inhibit SARS-CoV-2 and *E. coli* DNA gyrase.

## 1. Introduction

The emergence of resistant strains of the malaria parasite has necessitated the continued search for other effective, safe, and cheap antimalarial agents. The resistance of the malaria parasite to chloroquine and other anti-malarials has driven scientists into an intensive search for more effective agents against the scourge. Currently, there are reports of parasite resistance to the newly developed Artemisinin in some regions [1]. Additionally, the WHO warns that COVID-19 disruption to anti-malaria programs could cause deaths in Sub-Saharan Africa from malaria to double [2]. Thus, there is an urgent need for increased efforts in antimalarial drug discovery especially in Africa [3].

1,3,5-Triazine(*s*-triazine) analogs demonstrate a wide range of biological applications such as antimalarial, antimicrobial, antiviral, anticancer, antituberculosis, anti-HIV, antileishmanial, anti-inflammatory agents, insecticidal, and herbicidal [4,5,6,7,8,9,10]. Moreover, they are mostly used in the pharmaceutical, plastic, textile, and rubber industries [11,12,13]. They have also been used as dyestuffs, optical bleaches, explosives, and surface-active agents [14,15,16,17]. Additionally, cycloguanil, a derivative of 1,3,5-trazine, has been noted to be a cyclic metabolite of the antimalarial drug proguanil [18].

The *s*-triazine core has been applied in the development of less toxic bioactive compounds [19]. Consequently, melamine (2,4,6-triamino-*s*-triazine) based dendrimers are widely used in cancer and in many biological treatments [20]. Additionally, in the field of neuroleptics, *s*-triazines showed the most potent antimethamephetamine action with less toxicity than chlorpromazine [21]. Gupta and co-workers have reported thioether-linked *s*-triazine to 1,2,4-triazine derivative bearing piperidine substituent as potent against *Leishmania donovani* with less toxicity compared with standard pentamidine and sodium stibogluconate, respectively [22]. Comparison of *s*-triazine based drugs with other non-triazine based drugs has shown that *s*-triazine based drugs are more potent [19].

The literature has shown that *s*-triazine has been combined with other heterocycles with the aim of developing more potent drug-like molecules [23,24,25,26,27]. However, there is no report of *s*-triazine scaffolds containing lanthanides. In recent years, lanthanides and lanthanide compounds have attracted a great deal of interest because they have applications in medicinal inorganic chemistry and in material science [28]. In medicine, lanthanide complexes are exploited as contrast agents for magnetic resonance imaging (MRI) and as radiotherapeutic drugs [28,29]. Due to the catalytic, magnetic, and luminescent properties of lanthanide complexes with organic ligands, they have found application in electroluminescent devices and diodes, lasers, cathode ray tubes, sensors, dosimeters, biological fluoro-immunoassays, imaging agents, organic light emitting diodes (OLEDS), display application, decoration purposes, and telecommunication [30,31]. Lanthanide complexes with desired functions have been designed and synthesized using different kinds of organic ligands [32,33]. Moreover, lanthanides have an interesting, but not well understood, biological role in living organisms as trace elements [34]. Our group recently reported neodymium(III) and thallium(III) complexes of N-benzothiazol-2-yl)-4-chlorobenzenesulphonamide as potential antibacterial agents [35]. Recently, Taha et al. (2011) [36] reported that Nd, Dy, Sm, Pr, Gd, Tb, and Er complexes with bis-(salicylaldehyde)-1,3-propylenediimine Schiff base ligand possessed high antibacterial activity against *Shigella dysenteriae*, *Pseudomonas aeruginosa*, and *Proteus vulgaris* (Gram-negative bacteria).

In view of the noted biological properties of *s*-triazines and lanthanide(III) complexes, we synthesized and characterized 2,4,6-Tris(4-carboxybenzimino)-1,3,5-triazine (MT) and its trinuclear Dy(III), Er(III), and Gd(III) salen capped complexes. Their in vitro antimicrobial, in vivo antimalarial activities, and acute toxicity were also investigated. Additionally, in silico studies were carried to have a view of the interaction of the synthesized compounds with SARS-CoV-2 (6Y84) and *E.coli* DNA gyrase (5MMN).

## 2. Results and Discussion

**Scheme 1:**
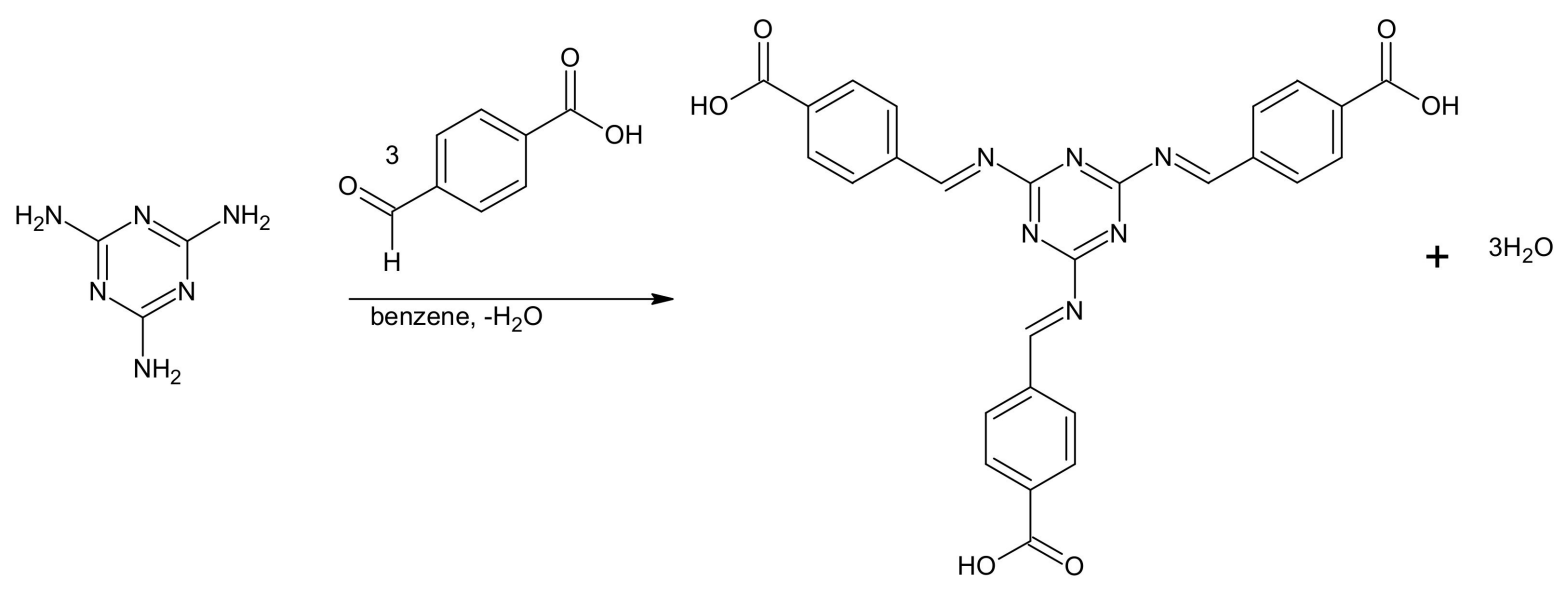
Synthesis of MT.

2,4,6-Tris(4-carboxybenzimino)-1,3,5-triazine (MT) was synthesized by the reaction of 2,4,6-triamino-1,3,5-triazine and 4-carboxybenzaldehyde (Scheme 1). MT was identified using FTIR, ^I^H, and ^13^C-NMR elemental analysis. It is soluble in acetone, chloroform, ethylacetate, DMF, and DMSO.

**Scheme 2:**
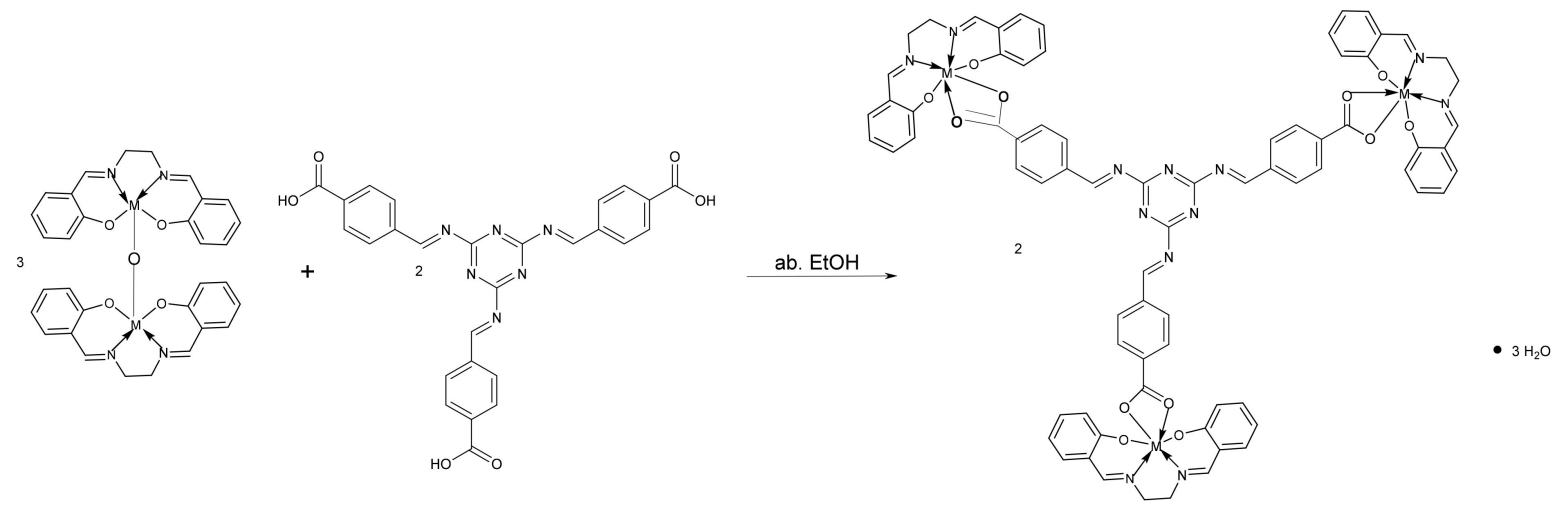
Synthesis of the tripodal trinuclear complexes; M = Dy, Er, Nd.

[Dy/Er/Gd(salen)]_2_O were used as Ligand complexes (LC) because they were able to coordinate to MT. The reaction of the LCs with MT gave rise to the tripodal trinuclear complexes, [{Dy/Er/Gd(salen)}_3_(MT)].3H_2_O (Scheme 2). These tripodal trinuclear complexes are the first examples of *s*-triazine based trinuclear complexes bridged to the Dysprosium(III), Erbium(III), and Gadolnium(III) centers by COO-. All complexes are stable at room temperature and soluble in DMSO and DMF but insoluble in water. The analytical data of MT and its trinuclear complexes are in good agreement with the proposed molecular formula as shown in Table 1. Molar conductivity measurements in methanol at room temperature show that the compounds are non-electrolytes [37].

### 2.1. Electronic Spectra

The UV/Vis absorption spectra of MT and its complexes (10^−4^ moldm^−3^) were carried out in methanol at room temperature, see Table 2 and Figure S1. The spectra are shown in supporting documents. The absorption band of MT showed two bands at 233 and 291 nm assigned to π–π* transitions of the benzene rings. In the Ln complexes, these bands are red shifted, supporting the coordination of MT to the Ln ions (Figures S2–S4).

### 2.2. Infrared Spectra

The relevant stretching frequencies of MT and its Ln(III) complexes are shown in Table 3, Figures S5–S8. The absorption band due to the carboxylic acid C=O was observed at 1674 cm^−1^ in MT [38,39]. This band shifted to higher frequencies of about 22–28 cm^−1^ in the complexes, suggesting coordination of the ligand complexes via the carboxylic acid C=O of MT. This was further supported by the vibrations of the COO^−^ group observed at 1410–1412 cm^−1^ in the complexes and at 1391 cm^−1^ in the ligand [40]. The IR spectrum of MT showed two medium bands for C=N(a) and C=N(b) at 1501 and 1573 cm^−1^, respectively. However, in the complexes, three bands were observed: C=N(a) bands at 1592–1596 cm^−1^, C=N(b) bands at 1628–1636 cm^−1^, and C=N(c) bands at 1536–1545 cm^−1^ respectively. The C=N(a) and C=N(b) stretching vibration in the complexes shifted to higher wavenumbers in comparison to the same transition in the ligand indicating delocalization of the double bond of the tripodal Schiff base in coordinating with the ligand complexes. While the C=N(c) band, which was absent in the tripodal Schiff base ligand but present in the complexes, confirms that the ligand complexes were actually capped to the tripod. Similar observation has been made in literature [38,39,40]. Bands in the range of 579–571 cm^−1^ in the tripodal–trinuclear complexes were assigned to **ν** (Ln–O) [36,41] while bands in the range of 489–437 cm^−1^ were assigned to ν (Ln–N) [42].

### 2.3. ^1^H and ^13^C-NMR Spectra

The ^1^H and ^13^C-NMR spectra of MT and its Ln(III) complexes are presented in Table 4 and Table 5 respectively. The ^1^H NMR spectrum of MT revealed a singlet peak at 10.17 ppm due to carboxylic proton (Figure S9). This peak disappeared in the complexes. The signal due to azomethine protons was observed between 9.77 and 8.29 ppm in the compounds. The signals in the range of 6.25–7.99 ppm in the compounds were assigned to aromatic protons. The signal due to ethylene protons appeared only in Dy(III)MT at 5.82 ppm. The signal due to DMSO was observed at 2.46 ppm in Dy(III)MT (Figure S10). The spectra for Er(III)MT and Gd(III)MT showed only solvent peak while that of Dy(III)MT showed only three peaks, probably due to the extent of paramagnetism of the Ln (III) ions [42,43].

The ^13^ C-NMR of MT gave signal at 193.47 ppm attributed to carboxylic carbon (Figure S11) [44]. The signal due to azomethine carbon was observed at 165.75 and 167.47 ppm in MT [45]. The signal at 139.07 and 136.98 ppm in MT has been assigned to carbons on the triazine ring. Carbons on the benzene ring are present at 130.32 and 129.92 ppm in MT. The spectra of the complexes revealed only the peak due to DMSO because of their paramagnetic nature (Figure S12) [42,43].

### 2.4. In Vitro Antimicrobial Activity

The results of the in vitro antimicrobial screening carried out on the compounds are given in Table 6. Ciprofloxacin, Tetracycline, Gentamicin, and Fluconazole were used as positive control while sterile DMSO served as negative control. These drugs have been chosen because they have the same mechanism of action, which is by inhibiting nucleic acid synthesis [46]. The structures of these drugs are shown in Supplementary Materials (Figure S13). Ciprofloxacin (C_17_H_18_FN_3_O_3_) belongs to fluoroquinolnes and inhibits bacteria growth by preventing Deoxyribonucleic acid (DNA) synthesis before mitosis. Tetracycline (C_22_H_24_N_2_O_8_) inhibits the multiplication of bacteria by binding to a subunit of the ribosomes, thereby inhibiting protein and nucleic acid synthesis and consequent death of the bacterium [47,48]. Gentamycin (C_21_H_43_N_5_O_7_) belongs to the class of aminoglycosides and acts by preventing protein synthesis, thereby inhibiting the synthesis of nucleic acids (DNA replication or RNA synthesis) and causes death of the bacterium. Fluconazole is an antifungal drug (C_13_H_12_F_2_N_6_O) and belongs to synthetic triazoles. Fluconazole inhibits fungal cytochrome P–450, an enzyme responsible for fungal sterol synthesis, thereby causing fungal cell walls to weaken [48].

The results obtained (Table 6) show that the compounds inhibited the growth of *Bacillus cereus*, *Staphylococcus aureus*, *Pseudomonas aeruginosa*, *Escherichia coli*, *Candida albicans*, and *Aspergillus niger* with inhibition zone diameter (IZD) in the range of 2–5, 1–15, 1–12, 2–8, 2–31, and 2–21 mm respectively. This reflects that the compounds exhibit higher activity against fungi (*Candida albicans* and *Aspergillus niger*) relative to the bacteria strains used. Among the test bacteria, the compounds were most active against *Staphylococcus aureus* followed by *Pseudomonas aeruginosa*. It was observed from the results (Table 6) that the activity of MT is higher than that of the trinuclear complexes. Hence, it could be inferred that the activity of the trinuclear complexes was not enhanced after anion coordination.

The inhibition zone diameter (IZD in mm) of the controls is displayed in Table S1. From Table S1, the inhibition zone diameters (IZD) of the controls are higher than that of the compounds.

The minimum inhibitory concentration (MIC) of the compounds and controls against *Bacillus cereus*, *Staphylococcus aureus*, *Pseudomonas aeruginosa*, *Escherichia coli*, *Candida albicans*, and *Aspergillus niger* are displayed in Table 7. From Table 7, the MIC of the compounds is found to be in the range of 50 for *Bacillus cereus*, 5.57–50 for *Staphylococcus aureus*, 6.27–50 for *Pseudomonas aeruginosa*, 7.3–50 for *Escherichia coli*, 2.6–50 for *Candida albicans*, and 2.3–50 for *Aspergillus niger*.

From Table 7, the MIC of the controls is found to be in the range of 1.40–6.25 μg/mL for *Bacillus cereus*, 0.70–6.25 μg/mL for *Staphylococcus aureus*, 0.63–6.25 μg/mL for *Pseudomonas aeruginosa*, 0.65–2.80 μg/mL for *Escherichia coli*, 0.64–2.50 μg/mL for *Candida albicans*, and 0.58–6.25 μg/mL for *Aspergillus niger*.

The MIC of MT against *Staphylococcus aureus* was 5.57 μg/mL while that of Gentamycin was 2.70 μg/mL. Among all the test compounds, MT was found to be the most active against *Candida albicans* and *Aspergillus niger* (MIC = 2.60 and 2.30 μg/mL, respectively). However, the standard antifungal drug Fluconazole was more active against *Candida albicans* and *Aspergillus niger* (MIC = 0.64 and 0.74 μg/mL, respectively) relative to MT.

### 2.5. Acute Toxicity

The acute toxicity test was recorded for MT alone as shown in Table S2. MT was selected because it showed higher sensitivity against the test micro-organisms. For mice administered with MT, no animal died within 24 h after administration (Table S2). This implies that MT is safe and has a wide range of effective doses (ED_50_).

### 2.6. In Vivo Antimalarial Studies

The suppressive antiplasmodic effect of methanolic solutions of MT samples on albino mice are displayed in Table S3 while Table 8 gives the percentage parasitemia inhibition.

Artesunate is an antimalarial drug with a molecular formular of C_19_H_28_O_8_ and was used as standard because of the presence of –COOH group in both Artesunate and MT. The structure is shown in Figure S14. Its mechanism of action is by inhibiting cytochrome oxidase of the malaria parasite surface membrane—food vacuole membrane and mitochondrial membrane—thereby blocking the supply of nutrients from the host cell cytoplasm [49]. It was observed from Table S3 that the effect of the samples on weight (Wt), PCV, and Hb of the infected mice treated did not show an orderly pattern of dose dependent effect. However, the effect was significant compared to the negative control. MT shows a significant dose dependent reduction of the effect of the sample on PCV, though in a negative manner. The effect of MT on Hb concentration shows the same effect as in PCV.

The results in Table 8 showed a general dose dependent significant parasitemia inhibition compared with the negative control with MT inhibition of 84.02% at 50 mg/kg close to the value (87.22%) of the standard drug—Artesunate at 5 mg/kg. At 25 mg/kg, MT gave 65.81%. This implies that MT can be used as an antimalarial drug after further tests.

### 2.7. In Silico Studies

COVID-19 is a deadly disease that has led to the loss of so many lives all over the world.

This disease is still ravaging the human race irrespective of the fact that some vaccines have been discovered. There is need for discovery of more drugs. It is therefore of paramount importance that drug-like molecules should target the SARS-CoV-2 6Y84 so as to identify compounds with high binding affinity, hence they could effectively combat COVID-19.

**Figure 1:**
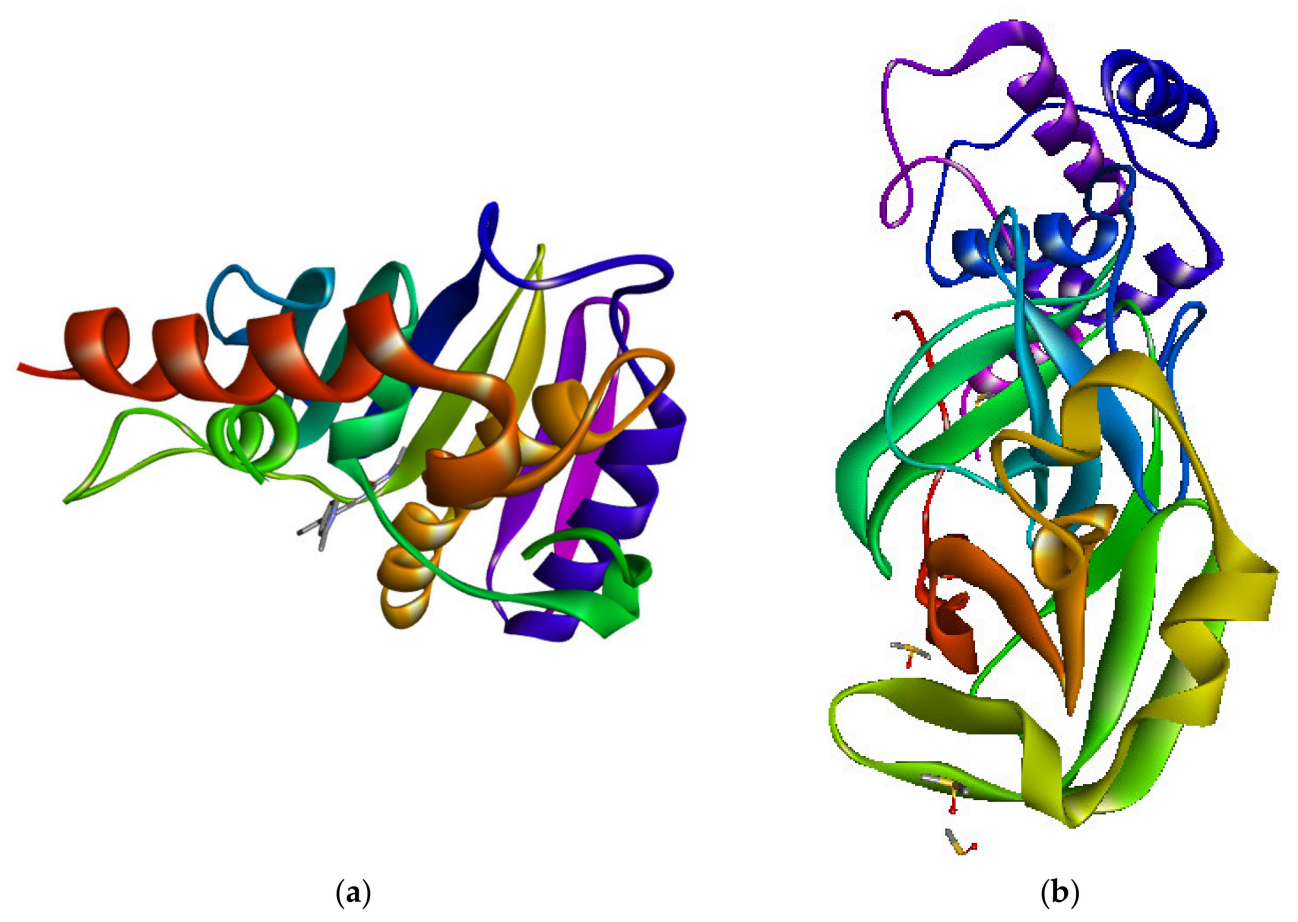
(**a**) *E. coli* DNA Gyrase B 24 kDa ATPase domain in complex with 1-ethyl-3-[8-methyl-5-(2-methyl-pyridin-4-yl)-isoquinolin-3-yl]-urea. (**b**) SARS-CoV-2 main protease with unliganded active site.

**Figure 2:**
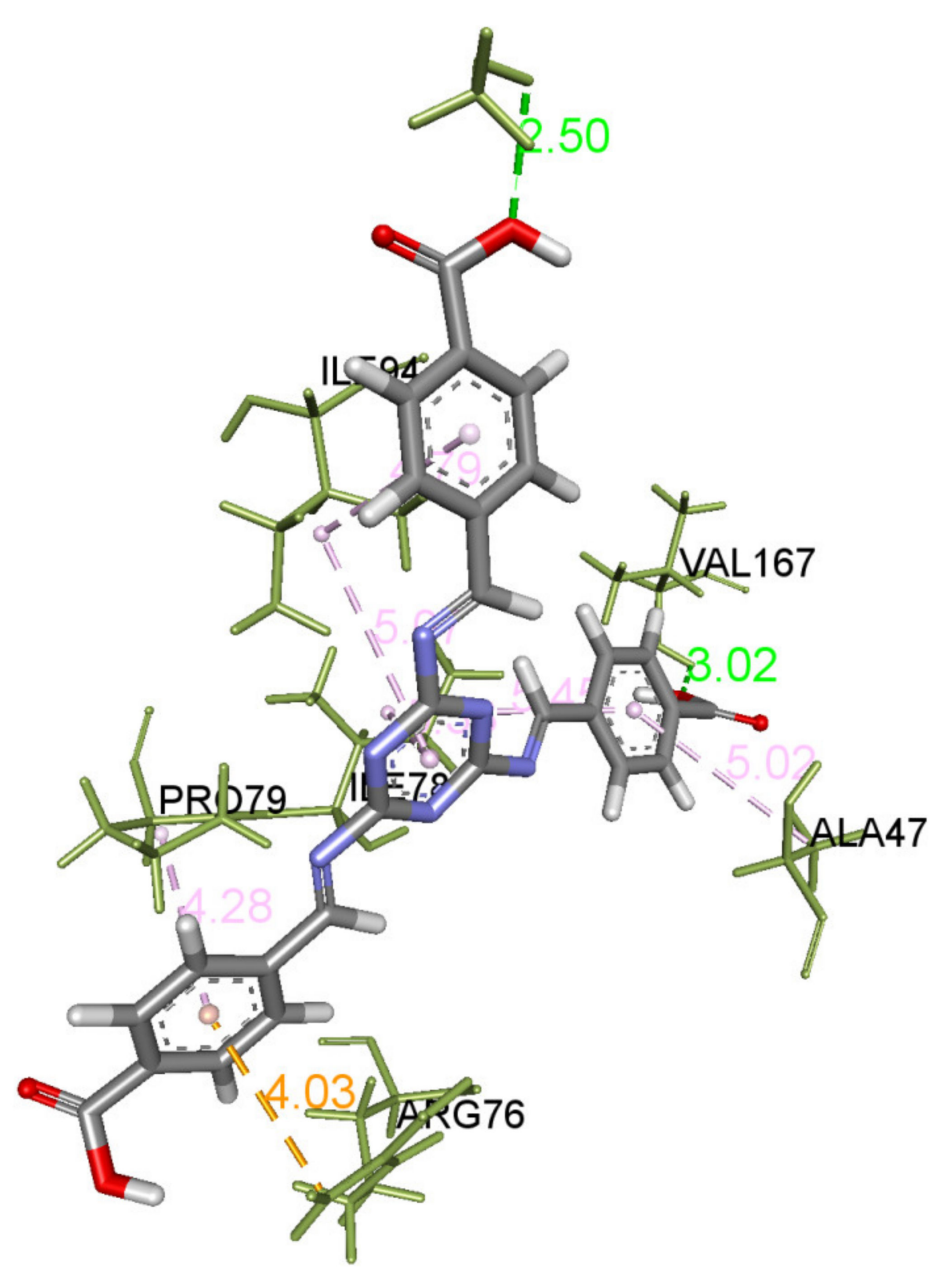
Binding interactions of MT with amino acid residues of *E. coli* DNA gyrase (5MMN). Key to bond type: orange dotted line = pi-cation bond; green dotted lines = H-bond; purple dotted line = pi-alkyl.

**Figure 3:**
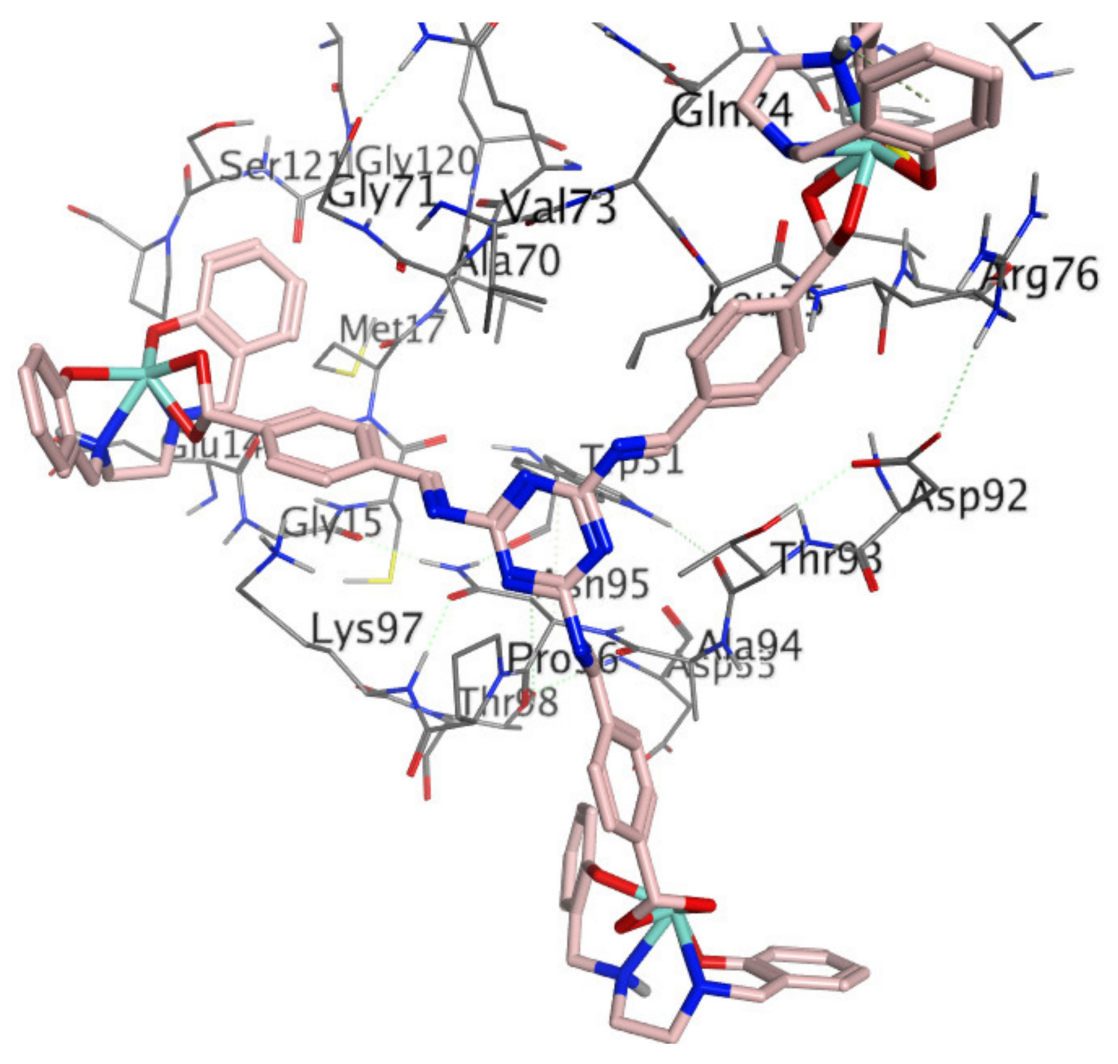
Binding pose of Er(III)MT in the active binding site of 6Y84.

**Figure 4:**
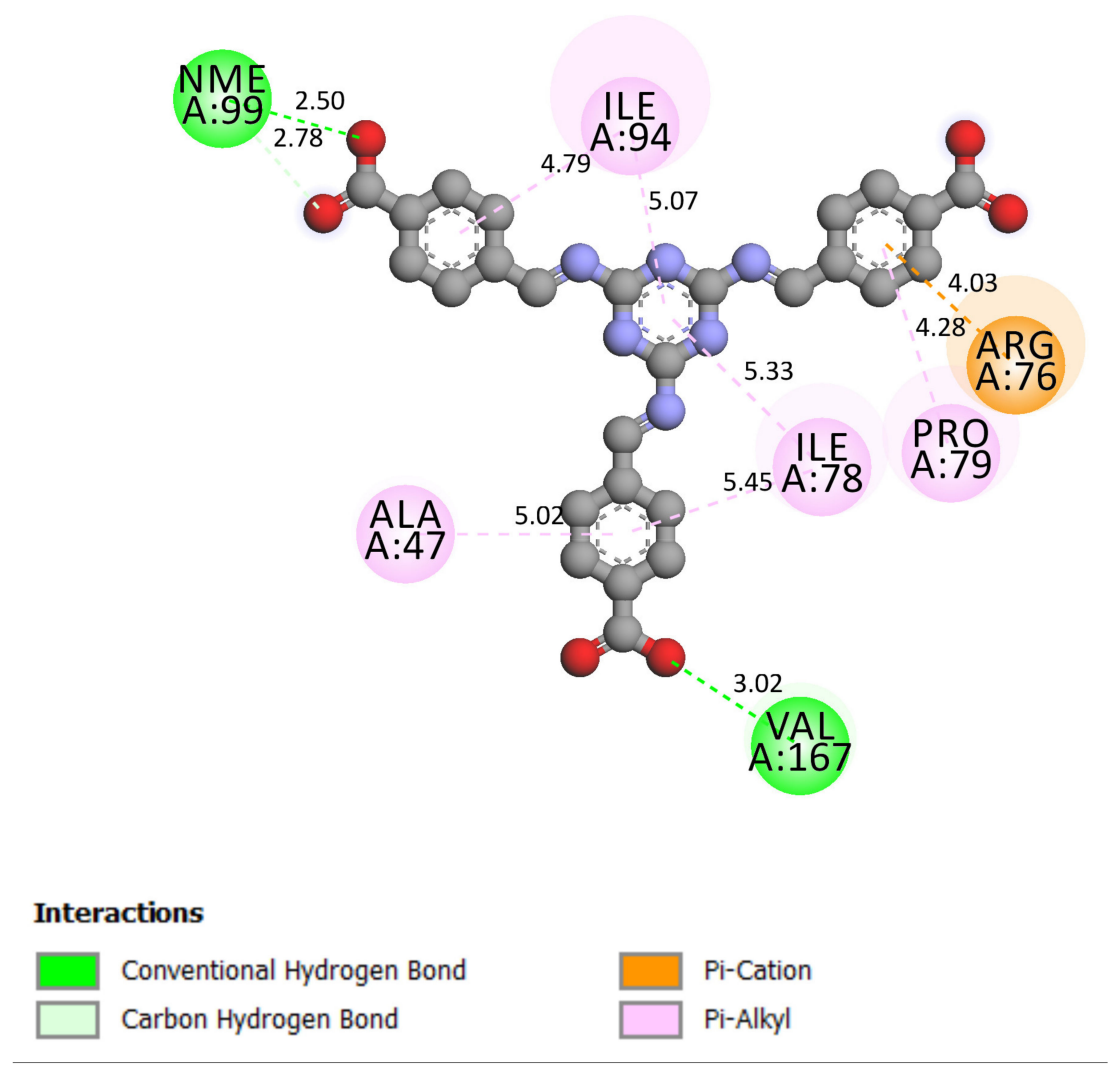
2D interaction of MT with the amino acid residues of *E. coli* DNA gyrase (5MMN).

Table 9 shows the binding free energies of the ligand and the complexes for both SARS-CoV-2 and antibacterial in silico studies. The binding energy reveals a strong binding affinity the ligand and the complexes have with the receptors. MT showed the highest binding affinity with *E. coli* DNA gyrase while the complex, Er(III)MT demonstrated the highest binding affinity to the SARS-CoV-2 receptor (Figure 1). To gain further insights into the nature and type of chemical interactions involved, the binding poses of these compounds in the active binding sites of the receptors were analyzed and are shown in Figure 2, Figure 3 and Figure 4. The atoms of the ligand, MT interacted with various amino acid residues of 5MMN. Significant interactions include H-bonding interaction between the O-atom of MT and VAL 160 through a distance of 3.02 Å. Pi-cation was observed between the pi-electrons of the phenyl group and ARG 76 (4.03 Å). Other amino acid residues involved include ALA 47, ILE 78 ILE 94, and PRO 79. Figure 3 depicts a well-fitted complex, Er(III)MT in the active binding site of 6Y84. The atoms of the complex interacted mostly with the amino acid residues through various hydrophobic interactions.

## 3. Materials and Methods

### 3.1. Materials

All the chemicals used were of analytical reagent grade, purchased from Zayo–Sigma and were used as supplied without further purification. The melting points of the compounds were determined using Fischer Jones melting point apparatus and were uncorrected. Molar conductance measurements were carried out using 10^−4^ mol/L solutions of the complexes in methanol at room temperature using a WTW-LF 90 conductivity meter. Electronic spectra (in methanol) were recorded on a UV-Vis 1800 SHIMADZU spectrophotometer. Infrared spectra of the compounds were performed using KBr discs on a Perkin–Elmer (Waltham, MA, USA) 100 series version 10.03.08 FTIR spectrophotometer. The ^1^H and ^13^C-NMR spectra of the compounds were recorded on a Bruker (Billerica, MA, USA) DPX 300 spectrometer in DMSO-d_6_ at 300.13 MHz and 75.47 MHz respectively. Elemental analysis for C, H, and N were carried out using an LECO–CHN–932 analyzer.

### 3.2. Synthesis of 2,4,6-Tris(4-carboxybenzimino)-1,3,5-triazine(MT)

The method reported by Uysal and Ucan (2009) [39] was adopted. Melamine (0.63 g, 0.005 mol) was dissolved in benzene (5 cm^3^) stirred for 1 h, then 4-carboxybenzaldehyde (2.25 g, 0.015 mole) was added and refluxed for 4 h. A white precipitate was obtained, filtered, and recrystallized from a mixture of methanol and water, dried and stored over CaCl_2_.

### 3.3. Synthesis of Ligand Complexes

The ligand complexes were prepared by addition of concentrated ammonia solution to a solution of [Dy/Er/Gd(salen)]_2_O in absolute ethanol, stirred at 50 °C until a pH of 12 was achieved [50,51].

### 3.4. Synthesis of [{Dy/Er/Gd(salen)}_3_(MT)].3H_2_O

[Dy/Er/Gd(salen)]_2_O (0.00037 mol) was suspended in hot absolute ethanol (25 cm^3^) and a solution of MT (0.13 g, 0.00025 mol) in absolute ethanol was added while stirring. The reaction mixture was boiled under reflux for 4 h. The light yellow solid formed was washed with water and dried over CaCl_2_ (Scheme 2).

### 3.5. In Vitro Antimicrobial Activity

The tripodal ligand and its trinuclear complexes were tested in vitro for their antimicrobial activities against American Type Culture Collection (ATCC) bacteria strains obtained from Rockville, MD, USA, by the Department of Microbiology, University of Nigeria, Nsukka while the fungi strains were isolated under clinical conditions. The typed bacteria culture was comprised of Gram-positive bacteria: *Staphylococcus aureus* (ATCC 6538P) and *Bacillus cereus* (ATCC 14579); Gram-negative bacteria: *Escherichia coli* (ATCC 6749) and *Pseudomonas aeruginosa* (ATCC 9027). The fungi strains used were *Candida albicans* and *Aspergillus niger*. The bacteria strains were tested for sterility on nutrient agar and then grown in nutrient broth at 37 °C for 24 h while the fungal strains were tested on Sabourand Dextrose Agar (SDA) and cultured in Sabourand Dextrose Liquid medium at 25 °C for 24 h. The overnight cultures were subsequently diluted and suspensions were made in normal saline and adjusted to 0.5 McFarland standards [52].

### 3.6. Antimicrobial Assay

The antimicrobial activities of all the synthesized compounds were determined by the agar cup diffusion technique [53]. The nutrient agar and SDA plates were inoculated with 0.1 mL broth culture of the test bacteria or fungi. Using a sterile cork borer, wells (5 mm in diameter and 2.5 mm deep) were bored into the inoculated agar. Fresh stock solutions (1000 μg/mL) of the synthesized compounds were prepared in DMSO. The stock solution was further diluted with sterilized distilled water to 12.5, 25, and 50 μg/mL for antimicrobial evaluation. The wells were filled with 100 µL of the test compounds by means of a sterile micropipette. Standard antibiotics, namely Ciprofloxacin, Tetracycline, Gentamycin, and Fluconazole were used as positive control while sterile DMSO served as negative control. Subsequently, 12.5, 6.25, and 3.125 μg/mL of each positive control were prepared in DMSO. The bacteria plates were incubated at 37 °C for 24 h while fungal plates were incubated at 25 °C for 24 h. Inhibition zone diameter (IZD) around each well was measured in millimeters and recorded. The graph of IZD^2^ against the log of concentration was plotted for each plate containing a specific compound and a micro-organism. The anti-log of the intercept on *x*-axis is the MIC.

### 3.7. Determination of Acute Toxicity (LD_50_)

All experiments involving the use of mice were conducted in compliance with NIH guidelines for care and use of animals [54]. MT was used for the test because it showed higher sensitivity against the tested micro-organisms. The oral acute toxicity of the ethanolic solutions of the samples were estimated in albino mice (100–250 g) by upper-level lethal dose (LD_50_) described by Lorke’s method [55]. A total of four mice of both sexes were employed and an acclimatization period of 24 h was allowed. The samples were weighed and dissolved in 3% ethanol. The ethanolic solutions of the samples were administered orally at doses of 1000, 1600, 2900, and 5000 mg/kg. The animals were monitored for 24 h and the number of deaths per group recorded. Then, the mice were observed continuously for one hour after treatment, intermittently for three hours, and thereafter over a period of 24 h. The mice were observed for gross behavioral changes such as feeding, hair erection, lacrimation, mortality, and other signs of toxicity manifestation. The mice were given access to food and clean water during the study.

### 3.8. In Vivo Studies

The in vivo antimalarial assay was done based on a 4-day suppressive test using mice. The evaluation of the antimalarial activity against the methanolic solutions of the samples and Artesunate sensitive *Plasmodium berghei* (NK 65) was carried out according to a standard protocol of Peter’s 4-day suppressive test [56]. Each of the 12 healthy experimental mice was inoculated intraperitoneally on the first day (Day 1). The infected mice were weighed and randomly divided into four groups of three mice each and four hours post inoculation were treated orally thereafter 24, 48, and 72 h post inoculation. For groups 1 and 2, 25 mg of sample/kg of mouse and 50 mg of sample/kg of mouse was administered orally for four consecutive days. For group 3, 5 mg of Artesunate/kg of mouse was administered while group 4 was given 5 mL of distilled water/kg of mouse for four consecutive days. On day 4 post inoculation, one drop of blood was taken from the tail of each experimental mouse and smeared on a microscope slide to make a thin film [57]. The thin films were fixed with methanol, stained with 10% Giemsa solution at pH 7.2 for 10 min, and examined microscopically. Parasitemia level was determined by counting the number of parasitized erythrocytes out of 100 erythrocytes per field in 4 random fields under a light microscope at magnification (×100) while average percentage parasitemia suppression was determined by comparing the parasitemia in the control group with the treated group.

$$\mathrm{Average}\;\mathrm{percentage}\;\mathrm{suppression}=pc-\frac{pt}{pc}\times 100$$

where *pc* = average parasitema in the control group, *pt* = average parasitemia in the treated group [58].

### 3.9. In Silico Study

The 3D structures of the receptors of SARS-CoV-2 (6Y84) and *E. coli* DNA gyrase (5MMN) as shown in Figure 1 were retrieved from the Protein Data Bank. The 3D structures of the ligand and complexes drugs drawn using MarvinSketch 17.2.6.0 were energy minimized and docked into the active binding sites of these receptors. Docking protocols were validated by reproducing the PDB crystal structures in silico. The various binding poses produced were analyzed with Discovery Studio vs16.1.0.15350.

DNA gyrase, a type II topoisomerase, is found in all bacteria. They consist of two subunits: GyrA and GryB of *E. coli* gyrase. These enzymes are responsible for catalyzing topological changes in DNA and have proved to be drug targets for therapeutic agents.

## 4. Conclusions

A tripodal Schiff base ligand derived from 2,4,6-triamino-1,3,5-triazine and its novel trinuclear Dy(III), Er(III), and Gd(III) salen capped complexes were synthesized and characterized based on various physico-chemical and spectral studies. The ligand was found to be tripodal and to coordinate to the ligand complexes via the carboxylic group. In vitro antimicrobial tests indicated that the tripodal ligand, MT is more active against the test micro-organisms relative to the trinuclear complex. Acute toxicity studies reveal that MT is safe and has a wide range of effective doses (ED_50_). In vivo antimalarial studies indicate that MT could serve as an effective antimalarial agent since it has parasitemia inhibition of 84.02% at 50 mg/kg and 65.81% at 25 mg/kg close to the value (87.22%) of the standard drug—Artesunate at 5 mg/kg. The chemical interactions of our synthesized ligand and complexes with SARS-CoV-2 and DNA gyrase revealed multiple effective bonding interactions. Through docking simulation, the binding energy calculated for the two drug targets showed active molecules with the ability to inhibit SARS-CoV-2 (COVID-19) and DNA gyrase.

## Figures and Tables

**Table 1:** Elemental and physical data of 2,4,6-Tris(4-carboxybenzimino)-1,3,5-triazine(MT) and its Ln(III) complexes.

Compound	Color	Λ_m_ (Ω^−1^cm^2^mol^−1^)	Yield g (%)	M.p. (°C)	Elemental Analysis % Calc. and Found					
					C		H		N	
					Calc.	Found	Calc.	Found	Calc.	Found
C_27_H_18_O_6_N_6_ (MT)	White	-	(2.25) 86.21	346 ^a^	62.07	61.95	3.45	3.40	16.09	15.90
C_75_H_57_N_12_O_12_Dy_3_.3H_2_O (Dy(III)MT)	Yellow	4.60	(0.27) 58.70	325 ^a^	48.43	48.30	3.39	3.50	9.04	8.80
C_75_H_57_N_12_O_12_Er_3_.3H_2_O (Er(III)MT)	Yellow	6.70	(0.28) 59.57	324 ^a^	48.08	48.10	3.37	3.50	8.97	8.60
C_75_H_57_N_12_O_12_Gd_3_.3H_2_O (Gd(III)MT)	Yellow	18.40	(0.30) 65.22	352 ^a^	48.86	48.90	3.42	3.60	9.12	8.90

^a^ = decomposition temperature.

**Table 2:** Electronic absorption data of 2,4,6-tris(4-carboxybenzimino)-1,3,5-triazine (MT) and its Ln(III) complexes.

Compound	λ_max_		ε × 10^3^ (mol^−1^dm^3^cm^−1^)	Band Assignment
	nm	cm^−1^		
MT	233	42,918	2.88	π–π*
	291	34,364	1.82	π–π*
Dy(III)MT	348	28,736	10.9	n–π*
Er(III)MT	347	28,818	9.72	n–π*
Gd(III)MT	350	28,571	9.82	n–π*

**Table 3:** IR band assignments (cm^−1^) for 2,4,6-tris(4-carboxybenzimino)-1,3,5-triazine (MT) and its Ln(III) metal complexes.

Compound	νC-H ar	νC=O	νC=N	νC–C	νCOO^−^	νC–N	νLn–O	νLn–N
MT	-	1674(s)	1501(m) a 1573(m) b	1422(m)	1391(m)	1168(m)	-	-
Dy(III)MT	3129(sh)	1698(m)	1594(m) a 1631(s) b 1542(m) c	1475(m) 1448(m)	1410(s)	1151(m) 1125(m)	579(m)	489(m) 437(m)
Er(III)MT	3158(w)	1696(m)	1596(s) a 1632(s) b 1543(s) c	1477(m)	1410(s)	1151(m) 1125(w)	575(m)	439(m)
Gd(III)MT	3125(m)	1702(m)	1592(m) a 1636(m) b 1540(s) c	1483(m)	1412(s)	1153(m)	571(m)	437(m)

Where a = from triazine ring; b = from azomethine linkage; c = from salen.

**Table 4:** ^1^H-NMR data of 2,4,6-tris(4-carboxybenzimino)-1,3,5-triazine (MT) and its Ln(III) Complexes (ppm).

Compound	OH_Carboxylic_	CH=N	H_aromatic_	CH_2_=CH_2_	DMSO
MT	10.17(1H,s)	9.77(1H,s) 8.29(1H,s)	7.99–6.25(4H,m)	-	2.50
Dy(III)MT	-	-	7.65(4H,m)	5.82(4H,s)	2.46
Er(III)MT	-	-	-	-	-
Gd(III)MT	-	-	-	-	-

**Table 5:** ^13^C-NMR data of 2,4,6-tris(4-carboxybenzimino)-1,3,5-triazine (MT) and its Ln(III) Complexes (ppm).

Compound	Carboxylic Carbon	Azomethine Carbon	Carbons on Triazine Ring	Aromatic Carbons	DMSO Peak
MT	193.47	167.47, 165.75	139.07, 136.98	130.32, 129.92	39.89
Dy(III)MT	-	-	-	-	39.58
Er(III)MT	-	-	-	-	-
Gd(III)MT	-	-	-	-	-

**Table 6:** Inhibition zone diameter (IZD in mm) of the compounds against typed strains (ATCC CULTURES) micro-organisms.

50 μg/mL						
Compound	*B. c* (ATCC 14579)	*S. a* (ATCC 6538P)	*P. a* (ATCC 9027)	*E. c* (ATCC 6749)	*C. a*	*A. n*
**MT**	5	15	12	6	31	21
**Dy(III)MT**	5	1	1	3	2	15
**Er(III)MT**	2	2	5	5	3	14
**Gd(III)MT**	3	9	2	8	2	12
**25 μg/mL**						
**MT**	-	10	7	2	19	15
**Dy(III)MT**	-	-		-	-	2
**Er(III)MT**	-	-	-	-	-	-
**Gd(III)MT**	-	3	-	4	-	-
**12.5 μg/mL**						
**MT**	-	5	3	3	6	10
**Dy(III)MT**	-	-	-	-	-	-
**Er(III)MT**	-	-	-	-	-	-
**Gd(III)MT**	-	-	-	-	-	-

Key: *B. c* = *Bacillus cereus*, *S. a* = *Staphylococcus aureus*, *P. a* = *Pseudomonas aeruginosa*, *E. c* = *Escherichia coli*, *C. a* = *Candida albicans*. *A. n* = *Aspergillus niger*, (-) = no zone of inhibition observed.

**Table 7:** Minimum inhibitory concentration (MIC) of the compounds and controls against test bacteria and fungi.

MIC (μg/mL)						
Compound	*B. c* (ATCC 14579)	*S. a* (ATCC 6538P)	*P. a* (ATCC 9027)	*E. c* (ATCC 6749)	*C. a*	*A. n*
**MT**	50	5.57	6.27	7.3	2.60	2.30
**Dy(III)MT**	50	50	50	50	50	25
**Er(III)MT**	50	50	50	50	50	50
**Gd(III)MT**	50	25	50	25	50	50
**Controls (μg/mL)**						
**T**	1.90	1.80	0.63	2.15	2.10	0.58
**F**	6.25	6.25	6.25	2.80	0.64	0.74
**CP**	1.50	0.70	0.92	0.65	2.00	6.25
**G**	1.40	2.70	0.71	2.60	2.50	0.64

Legend: T = Tetracycline, F = Fluconazole, CP = Ciprofloxacin, G = Gentamycin; *B. c* = *Bacillus cereus*, *S. a* = *Staphylococcus aureus*, *P. a* = *Pseudomonas aeruginosa*, *E. c* = *Escherichia coli*, *C. a* = *Candida albicans*, *A. n* = *Aspergillus niger*.

**Table 8:** Percentage of parasitemia inhibition.

Drug/Dose (mg/kg)	% Parasitemia	%Inhibition (PC)
MT 25	10.6667 ± 0.88192	65.81
MT 50	5.0000 ± 1.52753	84.02
Artesunate 5	4.00 ± 0.58	87.22
Dist. Water 5 mL/kg	31.33 ± 3.38	00.00

**Table 9:** Free binding energy of compounds against SARS-CoV-2 (6Y84) and *E. coli* DNA Gyrase (5MMN).

Compound	SARS-CoV-2. (6Y84)	*E. coli* DNA Gyrase (5MMN)
MT	−5.69	−7.03
Dy(III)MT	−7.30	−5.82
Er(III)MT	−7.98	−7.03
Gd(III)MT	−7.55	−6.59
CQ	−6.31	-
HCQ	−6.30	-
Cipro	-	−5.40

## Data Availability

Not applicable.

## Supplementary Materials

Figure S1: Electronic absorption spectrum of MT, Figure S2: Electronic absorption spectrum of Dy(III)MT, Figure S3: Electronic absorption spectrum of Er(III)MT, Figure S4: Electronic absorption spectrum of Gd(III)MT, Figure S5: Infrared Spectrum of MT, Figure S6: Infrared Spectrum of Dy(III)MT, Figure S7: Infrared Spectrum of Er(III) MT, Figure S8: Infrared Spectrum of Gd(III)MT, Figure S9: ^1^H-NMR Spectrum of MT, Figure S10: ^1^H-NMR Spectrum of Dy(III)MT, Figure S11: ^13^C-NMR Spectrum of MT, Figure S12: ^13^C-NMR Spectrum of Dy(III)MT, Figure S13: Structures of the drugs used as standard, Figure S14: Structure of Artesunate, Table S1: Inhibition Zone Diameter (IZD in mm) of the Controls, Table S2: Determination of Acute Toxicity (LD50), Table S3: Anti-plasmodic Effect of Samples on Albino mice.

## References

[1] WHO (2017). Artemisinin and Artemisinin-Based Combination Therapy Resistance. http://www.who.int/malaria/publications/atoz/artemisinin-resistanceapril2017/en/.

[2] WHO (2020). Malaria Could Make a Comeback Thanks to COVID-19. https://www.weforum.org/agenda/2020/04/malaria-treatment-rise-africa-coronavirus/.

[3] Fidock D.A., Rosenthal P.J., Croft S.L., Brun R., Nwaka S. (2004). Antimalarial Drug Discovery: Efficacy Models for Compound Screening. Nat. Rev. Drug Discov..

[4] Ojha H., Gahlot P., Tiwari A.K., Pathak M., Kakkar R. (2011). Quantitative structure activity relationship study of 2, 4, 6-trisubstituted-*s*-triazine derivatives as antimalarial inhibitors of *Plasmodium falciparum* dihydrofolate reductase. Chem. Biol. Drug Des..

[5] Modh R.P., Patel A.C., Chikhalia K.H. (2013). Design, synthesis, antibacterial, and antifungal studies of novel 3-substituted coumarinyl-triazine derivatives. Heterocycl. Commun..

[6] Machakanur S.S., Patil B.R., Badiger D.S., Bakale R.P., Gudasi K.B., Annie B.S. (2012). Synthesis, characterization and anticancer evaluation of novel tri-arm star shaped 1, 3, 5-triazine hydrazones. J. Mol. Struct..

[7] Lozano V., Aguado L., Hoorelbeke B., Marleen R., María-José C., Dominique S., Jan B., Ana S.-F., María-Jesús P.-P. (2011). Targeting HIV entry through interaction with envelope glycoprotein 120 (gp120): Synthesis and antiviral evaluation of 1, 3, 5-triazines with aromatic amino acids. J. Med. Chem..

[8] Moni S., Kuldeep C., Rahul S., Preeti V., Manish K.S., Abhisheak S., Suman G., Jitendra K.S., Jawahar L., Preeti C. (2013). Discovery of a new class of natural product-inspired quinazolinone hybrid as potent antileishmanial agents. J. Med. Chem..

[9] Chiara D., Massimo C., Margherita G., Roberto F., Simona S., Giovanni S., Rita M. (2010). Evaluation of in vitro anti-inflammatory activity of some 2-alkyl-4, 6-dimethoxy-1, 3, 5-triazines. J. Pharm. Pharmacol..

[10] Zhao H., Liu Y., Cui Z., Beattie D., Gu Y., Wang Q. (2011). Design, synthesis, and biological activities of arylmethylamine substituted chlorotriazine and methylthiotriazine compounds. J. Agric. Food. Chem..

[11] Uysal S., Koc Z.E. (2010). Synthesis and Characterization of Dendrimeric Melamine Cored [salen/salophenFe(III)] and [salen/salophenCr(III)] Capped Complexes and Their Magnetic Behaviors. J. Hazard. Mater..

[12] Wu J., Chen L., Fu T., Zhao H., Guo D., Wang X., Wang Y. (2018). New application for aromatic Schiff base: High efficient flameretardant and anti-dripping action for polyesters. Chem. Eng. J..

[13] Agathian K., Kannammal L., Meenarathi B., Kailash S., Anbarasan R. (2018). Synthesis, characterization and adsorption behavior of cotton fiberbased Schiff base. Int. J. Biol. Macromol..

[14] Naz A., Arun S., Narvi S.S., Alam M.S., Singh A., Bhartiya P., Dutta P.K. (2018). Cu(II)-carboxymethyl chitosan-silane schiff basecomplex grafted onnano silica: Structural evolution, antibacterial performance and dyedegradation ability. Int. J. Biol. Macromol..

[15] Al-Hamdani A.A.S., Balkhi A.M., Falah A., Shaker S.A. (2016). Synthesis and investigation of thermal properties of vanadyl complexes with azo-containing Schiff-base dyes. J. Saudi Chem. Soc..

[16] Lu F., Astruc D. (2018). Nanomaterials for removal of toxic elements from water. Coord. Chem. Rev..

[17] Diem H., Matthias G. (2006). Amino Resins. Ullmann’s Encyclopedia of Industrial Chemistry.

[18] Tripathi K.D. (2003). Essentials of Medical Pharmacology.

[19] Shah D., Modh R.P., Chikhalia K.H. (2014). Privileged s-triazines: Structure and pharmacological applications. Future Med. Chem..

[20] Hatfield S.E. (2007). Applications of Triazine Chemistry: Education, Remediation, and Drug Delivery. Ph.D. Thesis.

[21] Tobe A., Kobayashi T. (1976). Pharmacological studies on triazine derivatives V. Sedative and neuroleptic actions of 2-amino-4-(4-(2- hydroxyethyl)-piperazin-1-yl)-6- trifluoromethyl-s-triazine (TR-10). Jpn. J. Aerosp. Med. Psychol..

[22] Leena G., Naresh S., Aditya V., Saumya S., Suman G., Neena G., Prem M.S.C. (2010). Synthesis and biological evaluation of new [1,2,4] triazino[5,6-b] indol-3-ylthio-1,3,5-triazines and [1,2,4] triazino [5,6-b] indol-3-yl thio-pyrimidines against *Leishmania donovani*. Eur. J. Med. Chem..

[23] Hashmi S.Z. (2016). Synthesis of Pharmacologically Important s-Triazine Derivatives. J. Pharmacol. Res. Rev..

[24] Sonikan J., Pankaj K.J., Shalu S., Devarapalli K., Jaya D. (2020). Anticancer s-triazine derivatives: A synthetic attribute. Mini-Rev. Org. Chem..

[25] Guo F.J., Hyun S.B., Hiroyuki N., Jong-Dae L. (2018). O-Carboranylalkoxy-1,3,5-Triazine Derivatives: Synthesis, Characterization, X-ray Structural Studies, and Biological Activity. Molecules.

[26] Cascioferro S., Parrino B., Spanò V., Carbone A., Montalbano A., Barraja P., Diana P., Cirrincione G. (2017). 1,3,5-Triazines: A promising scaffold for anticancer drugs development. Eur. J. Med. Chem..

[27] Nie Z., Perretta C., Erickson P., Margosiak S., Lu J., Averill A., Almassy R., Chu S. (2008). Structure-based design and synthesis of novel macrocyclic pyrazolo[1,5-a] [1,3,5]triazine compounds as potent inhibitors of protein kinase CK2 and their anticancer activities. Bioorg. Med. Chem. Lett..

[28] Salehzadeh S., Nouri S.M., Keypour H., Bagherzadeh M. (2005). Synthesis of Gadolinium(III) and Samarium(III) Complexes of New Potentially Heptadentate(N_4_O_3_) Tripodal Schiff Base Ligands, and a Theoretical Study. Polyhedron.

[29] Casellato U., Tamburini S., Tomasin P., Vigato P.A., Botta M. (1996). Lanthanide(III) Complexes with a Podand Schiff Base Containing an N_4_O_3_ Coordination Site. Inorg. Chim. Acta.

[30] Mikhalyova E.A., Yakovenko A.V., Zeller M., Gavrilenko K.S., Lofland S.E., Addiso A.W., Pavilshchuk V.V. (2014). Structure, Magnetic and Luminescence Properties of Lanthanide Complexes Ln_2_(Salphen)_3_.H_2_O (Ln = Pr, Nd, Sm, Eu, Gd, Tb,Dy; H_2_Salphen = N,N^1^-Bis(salicylidene)-1,2-phenylenediamine). Inorg. Chim. Acta.

[31] Chen R.X., Gao T., Sun W.B., Li H.F., Wu Y.H., Xu M.M., Zou Y., Yan P.F. (2015). Salen Homonuclear and Heteronuclear Lanthanide(III) Complexes with Near-Infrared (NIR) Luminescence. Inorg. Chem. Comm..

[32] Liu H., Zhang Y., Feng W.X., Su P.Y., Shi G.X., Zhang Z., Fan D.D., Lu R., Lu X.Q., Wong W.K. (2013). Photo-luminescent Hetero-tetranuclear Zn_2_Ln_2_ (Ln = Nd, Yb, Er, Gd, Eu or Tb) Complexes Self-assembled from the Benzimidazole-based HL and bpe. Inorg. Chem. Comm..

[33] Chen C., Chen C., Yan P., Hou G., Li G. (2013). Structure and Electrochemistry of Salen Cerium(IV) Complexes Tuned by Multiform Counter Ions. Inorg. Chim. Acta.

[34] Oruma U.S., Ukoha P.O., Asegbeloyin J.N. (2014). Synthesis, Characterization and Biological Studies of S-1,3-Benzothiazol-2-ylthiophene-2-carbothioate and its Ce(IV) and Nd(III) Complexes. Asian J. Chem..

[35] Obasi L.N., Oruma U.S., Al-Swaidan I.A., Ramasami P., Ezeorah C.J., Ochonogor A.E. (2017). Synthesis, Characterization and Antibacterial Studies of N-(Benzothiazol-2-yl)-4-chlorobenzenesulphonamide and its Neodymium(III) and Thallium(III) Complexes. Molecules.

[36] Taha Z.A., Ajlouni A.M., Al-Hassan K.A., Hajazi A.K., Faiq A.B. (2011). Synthesis, Characterization, Biological Activity and Fluorescence Properties of Bis-(salicylaldehyde)-1,3-propylenediimine Schiff base Ligand and Its Lanthanide Complexes. Spectrochim. Acta Part A.

[37] Ali I., Wani W.A., Saleem K. (2013). Empirical formulae to molecular structures of metal complexes by molar conductance. Synth. React. Inorg. Met. Chem..

[38] Uysal S., Koc Z.E. (2016). Synthesis and Characterization of Dopamine Substitue Tripodal Trinuclear [(salen/salophen/salpropen) M] (M = Cr(III), Mn(III), Fe(III) ions) Capped S-triazine Complexes: Investigation of their Thermal and Magnetic Properties. J. Mol. Struc..

[39] Uysal Ş., Uçan H.I. (2009). The synthesis and characterization of melamine based Schiff bases and its trinuclear [salen/salophenFe (III)] and [salen/salophenCr (III)] capped complexes. J. Incl. Phenom. Macrocycl. Chem..

[40] Uysal Ş., Koç Z.E., Çelikbilek Ş., Uçan H.İ. (2012). Synthesis of star-shaped macromolecular schiff base complexes having melamine cores and their magnetic and thermal behaviors. Synth. Commun..

[41] Lekha L., Raja K.K., Rajagopal G., Easwaramoorthy D. (2014). Synthesis, spectroscopic characterization and antibacterial studies of lanthanide (III) Schiff base complexes containing N, O donor atoms. J. Mol. Struct..

[42] Bertini I., Luchinat C., Parigi G. (2002). Magnetic Susceptibility in Paramagnetic NMR. Prog. Nucl. Magn. Reson. Spectrosc..

[43] İşçi B., Uysal S. (2018). The synthesis and characterization of [M(salen/salophen/saldeta)][M=Cr(III), Mn(III) or Fe(III)] capped s-triazine cored tripodal trinuclear Schiff bases complexes. J. Incl. Phenom. Macrocycl. Chem..

[44] Silverstein R.M., Webster F.X., Kiemle D.J. (2005). Spectrometric Identification of Organic Compounds.

[45] Ukoha P.O., Oruma U.S. (2014). Synthesis and Antimicrobial Studies of N, N^1^-Bis(4- Dimethylaminobezylidene)ethane-1,2-diamine (DAED) and its Nickel(II) and Platinium(IV) complexes. J. Chem. Soc. Niger..

[46] Uchechukwu S.O., Pius O.U., Lydia R., Mohamed I.E., Lawrence N.O., Ponnadurai R., Klaus J. (2018). Synthesis, Characterization, Antimicrobial Screening, and Computational Studies of a Tripodal Schiff Base Containing Pyrimidine Unit. J. Heterocycl. Chem..

[47] Kostova I., Manolov I., Momekov G. (2004). Cytotoxic Activity of New Neodymium (III) Complexes of Bis-coumarins. Eur. J. Med. Chem..

[48] Wolters K. (2009). Clinical Pharmacology Made Incredibly Easy.

[49] Artesunate (2016). Artesunate Chemical Properties, Usage, Production. www.chemicalbook.com/ChemicalProductProperty_EN_CB3157307.htm.

[50] Kopel P., Sindelar Z., Klicka R. (1998). Complexes of Iron(III) Salen and Saloph Schiff Bases with Bridging Dicarboxylic and Tricarboxylic Acids. Transit. Met. Chem..

[51] Gembicky M., Boca R., Renz F. (2000). A Heptanuclear Fe(III)-Fe(III)6 System with Twelve Unpaired Electrons. Inorg. Chem. Comm..

[52] Cheesbrough M. (2006). District Laboratory Practice in Tropical Countries.

[53] Alli A., Ehinmidu J., Ibrahim Y. (2011). Preliminary phytochemical screening and antimicrobial activities of some medicinal plants used in Ebiraland. Bayero J. Pure Appl. Sci..

[54] Council N.R. (2010). Guide for the Care and Use of Laboratory Animals.

[55] Lorke D. (1983). A new approach to practical acute toxicity testing. Arch. Toxicol..

[56] Peters W. (1975). The four-day suppressive in vivo antimalarial test. Ann. Trop. Med. Parasitol..

[57] Saidu K., Onah J., Orisadipe A., Olusola A., Wambebe C., Gamaniel K. (2000). Antiplasmodial, analgesic, and anti-inflammatory activities of the aqueous extract of the stem bark of *Erythrina senegalensis*. J. Ethnopharmacol..

[58] Ukwe V.C., Epueke E.A., Ekwunife O.I., Okoye T.C., Akudor G.C., Ubaka C.M. (2010). Antimalarial activity of aqueous extract and fractions of leaves of Ageratum conyzoides in mice infected with Plasmodium berghei. Int. J. Pharm. Sci..

