# Supplementary material for: Synthesis, Biological and In Silico Studies of a Tripodal Schiff Base Derived from 2,4,6-Triamino-1,3,5-triazine and Its Trinuclear Dy(III), Er(III), and Gd(III) Salen Capped Complexes
Source: Molecules. 2021 Jul 20;26(14):4379. doi: 10.3390/molecules26144379 (PMC8304507; doi:10.3390/molecules26144379)
Supplement: Supplementary file 1 [file molecules-26-04379-s001.zip › molecules-1247548-supplementary.pdf]

Synthesis, Biological and *In silico* Studies of a Tripodal Schiff Base Derived from 2,4,6-Triamino-1,3,5-triazine and its Trinuclear Dy(III), Er(III) and Gd(III) Salen Capped Complexes.

Uchechukwu Susan Oruma[1], Pius Oziri Ukoha[1], Chiamaka Peace Uzoewulu[2], Joseph Chinedum Ndefo[3], Sabastine Chinweike Ugwuoke[4], Nkechinyere Nwanneka Ukwueze[2], Tochukwu Emmanuella Eze[2,4], Chinenye Lilian Ekowo[2], Florence Uchenna Eze[2] and Uchenna Vivian Obi[2,5] Sunday Nwankwo Okafor[6]* Chigozie Julius Ezeorah[2]*.

SUPPLEMENTARY MATERIALS

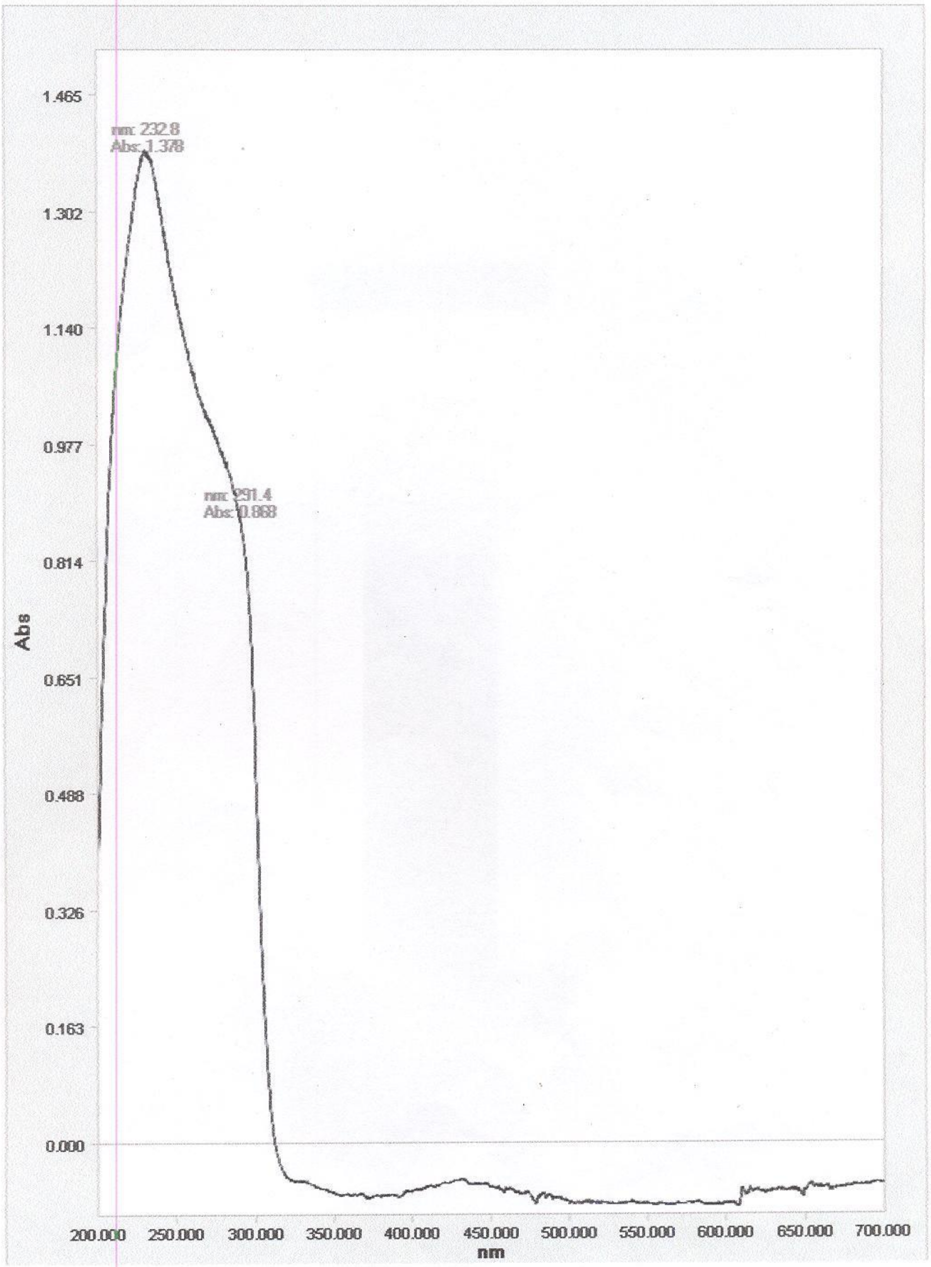

nm: 232.8
Abs: 1.378
nm: 291.4
Abs: 0.868
1.465
1.302
1.140
0.977
0.814
0.651
0.488
0.326
0.163
0.000
Abs
200.000
250.000
300.000
350.000
400.000
450.000
500.000
550.000
600.000
650.000
700.000
nm

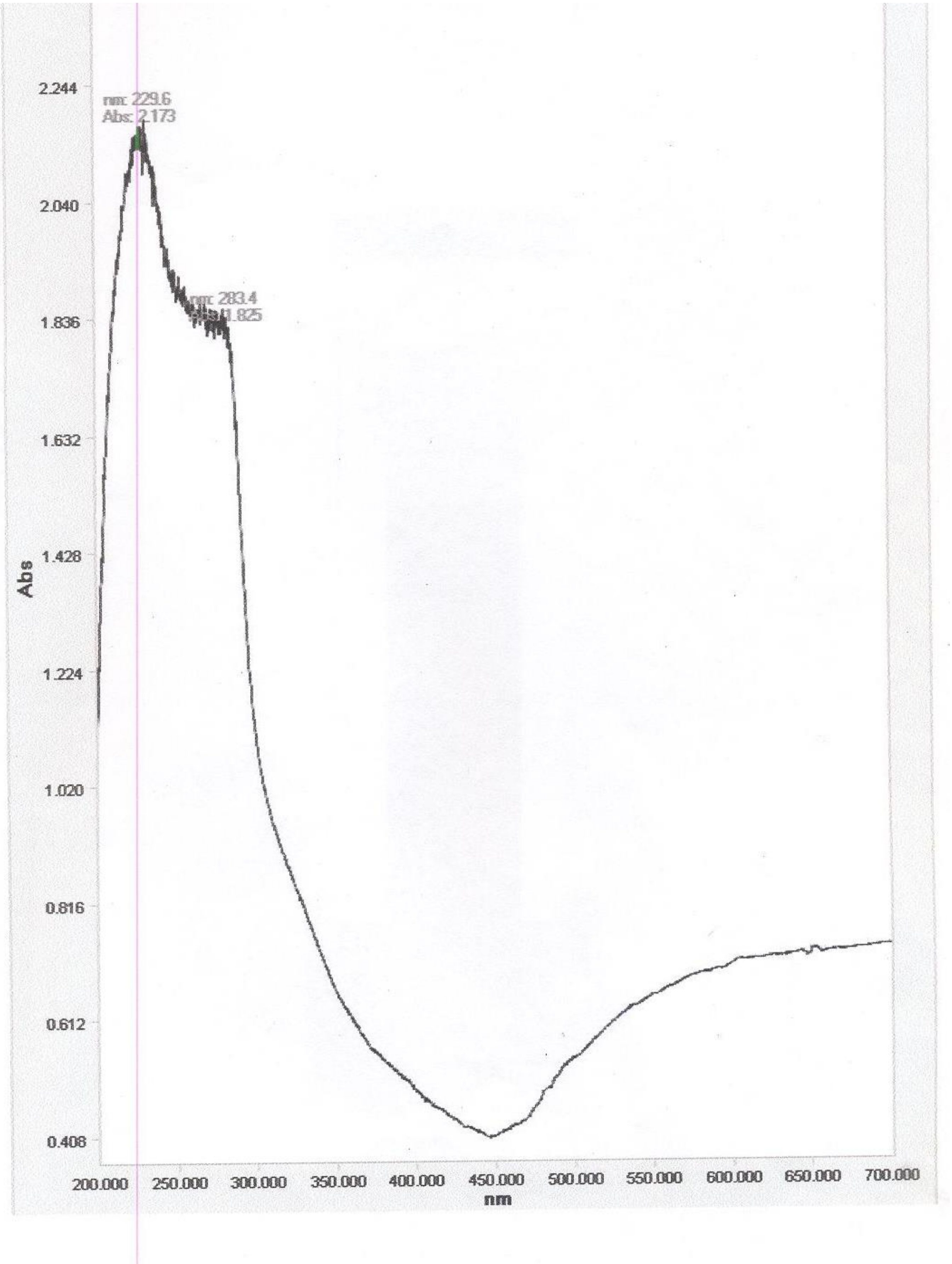

Figure S1: Electronic absorption spectrum of MT.

Spectrum Peak Pick Report

01/19/2016 01:55:10 PM

Data Set: MT2 - RawData

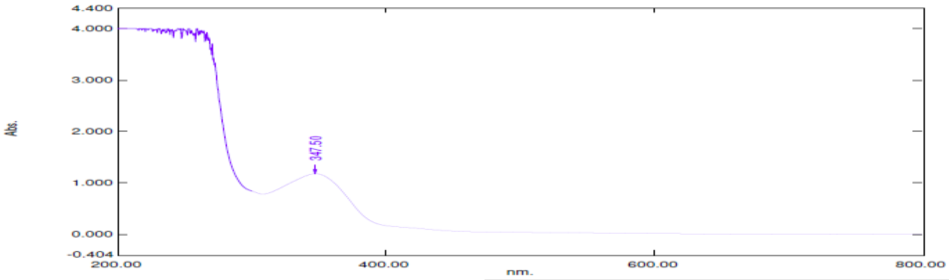

No.	P/V	Wavelength	Abs.	Description
1	⊕	347.50	1.177	347.50
2	⊕	308.00	0.779	

**Measurement Properties**	
Wavelength Range (nm.):	200.00 to 800.00
Scan Speed:	Fast
Sampling Interval:	0.5
Auto Sampling Interval:	Disabled
Scan Mode:	Single
**Instrument Properties**	
Instrument Type:	UV-1800 Series
Measuring Mode:	Absorbance
Slit Width:	1.0 nm
Light Source Change Wavelength:	340.8 nm
S/R Exchange:	Normal
**Attachment Properties**	
Attachment:	6-Cell
Number of cells:	6
**Sample Preparation Properties**	
Weight:	
Volume:	
Dilution:	
Path Length:	
Additional Information:	

Page 1 / 1

Figure S2: Electronic absorption spectrum of Dy(III)MT.

Spectrum Peak Pick Report

01/19/2016 01:57:59 PM

Data Set: MT3 - RawData

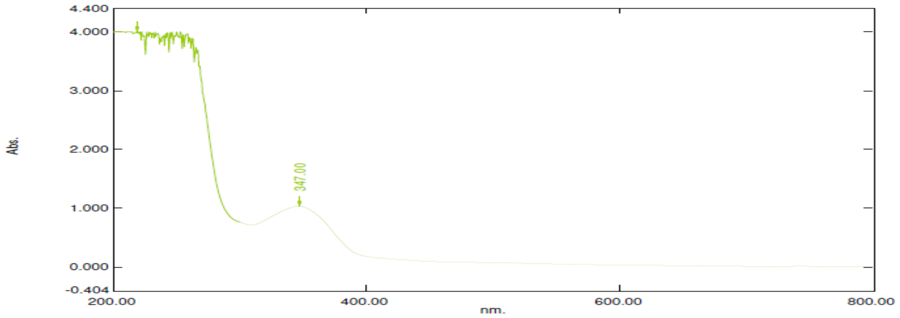

No.	P/V	Wavelength	Abs.	Description
1	⊕	347.00	1.036	347.00
2	⊕	218.50	4.000	
3	⊕	309.00	0.707	

**Measurement Properties**	
Wavelength Range (nm.):	200.00 to 800.00
Scan Speed:	Fast
Sampling Interval:	0.5
Auto Sampling Interval:	Disabled
Scan Mode:	Single
**Instrument Properties**	
Instrument Type:	UV-1800 Series
Measuring Mode:	Absorbance
Slit Width:	1.0 nm
Light Source Change Wavelength:	340.8 nm
S/R Exchange:	Normal
**Attachment Properties**	
Attachment:	6-Cell
Number of cells:	6
**Sample Preparation Properties**	
Weight:	
Volume:	
Dilution:	
Path Length:	
Additional Information:	

Page 1 / 1

Figure S3: Electronic absorption spectrum of Er(III)MT.

Spectrum Peak Pick Report

01/19/2016 02:02:42 PM

Data Set: MT4 - RawData

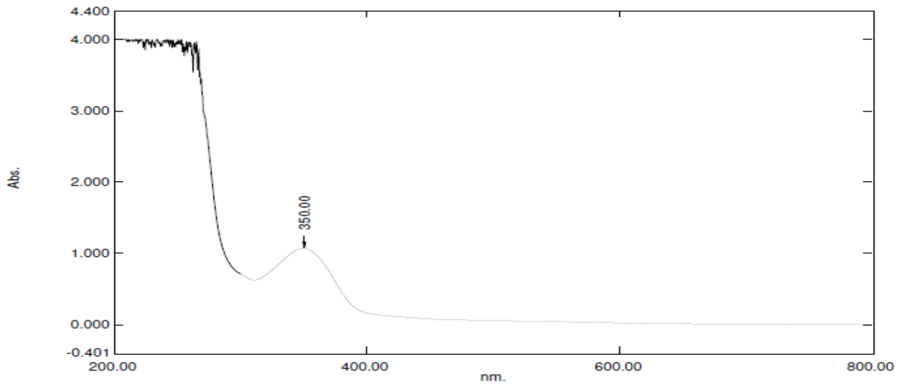

No.	P/V	Wavelength	Abs.	Description
1		350.00	1.070	350.00
2		310.00	0.624	

Measurement Properties
Wavelength Range (nm.): 200.00 to 800.00
Scan Speed: Fast
Sampling Interval: 0.5
Auto Sampling Interval: Disabled
Scan Mode: Single

Instrument Properties
Instrument Type: UV-1800 Series
Measuring Mode: Absorbance
Slit Width: 1.0 nm
Light Source Change Wavelength: 340.8 nm
S/R Exchange: Normal

Attachment Properties
Attachment: 6-Cell
Number of cells: 6

Sample Preparation Properties
Weight:
Volume:
Dilution:
Path Length:
Additional Information:

Page 1 / 1

Figure S4: Electronic absorption spectrum of Gd(III)MT.

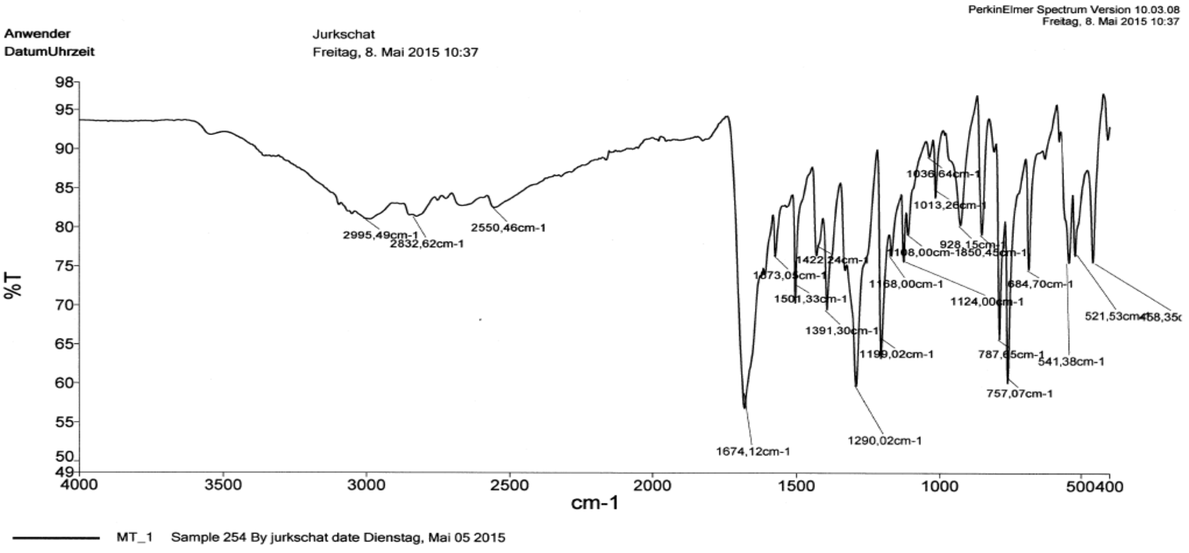

Figure S5: Infrared Spectrum of MT.

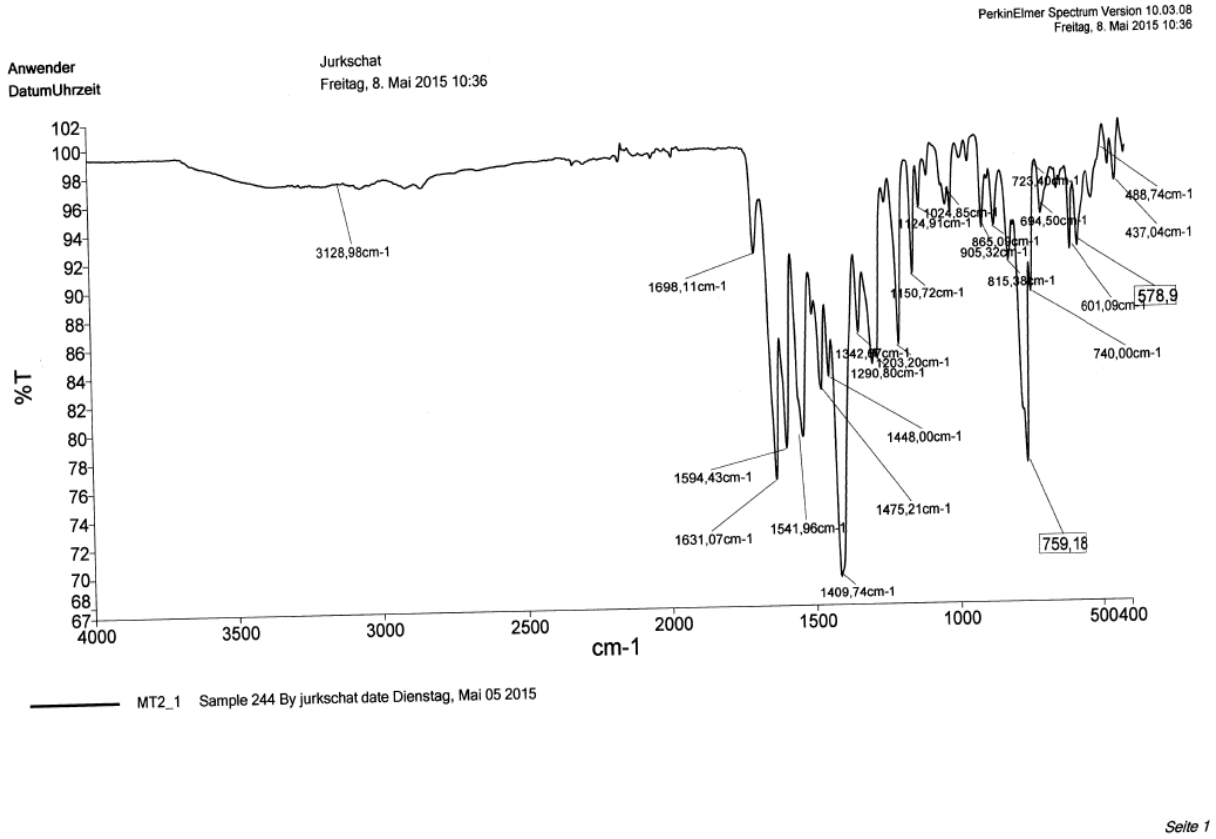

Figure S6: Infrared Spectrum of Dy(III)MT

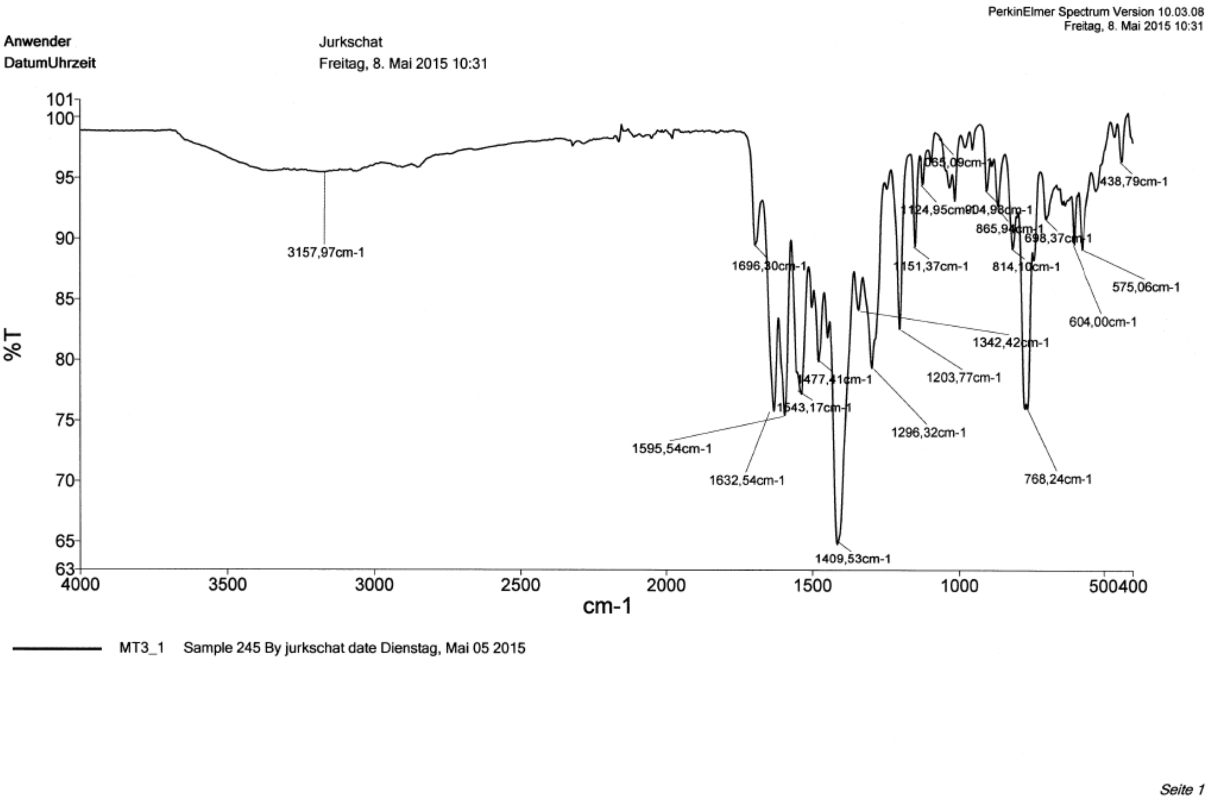

Figure S7: Infrared Spectrum of Er(III) MT

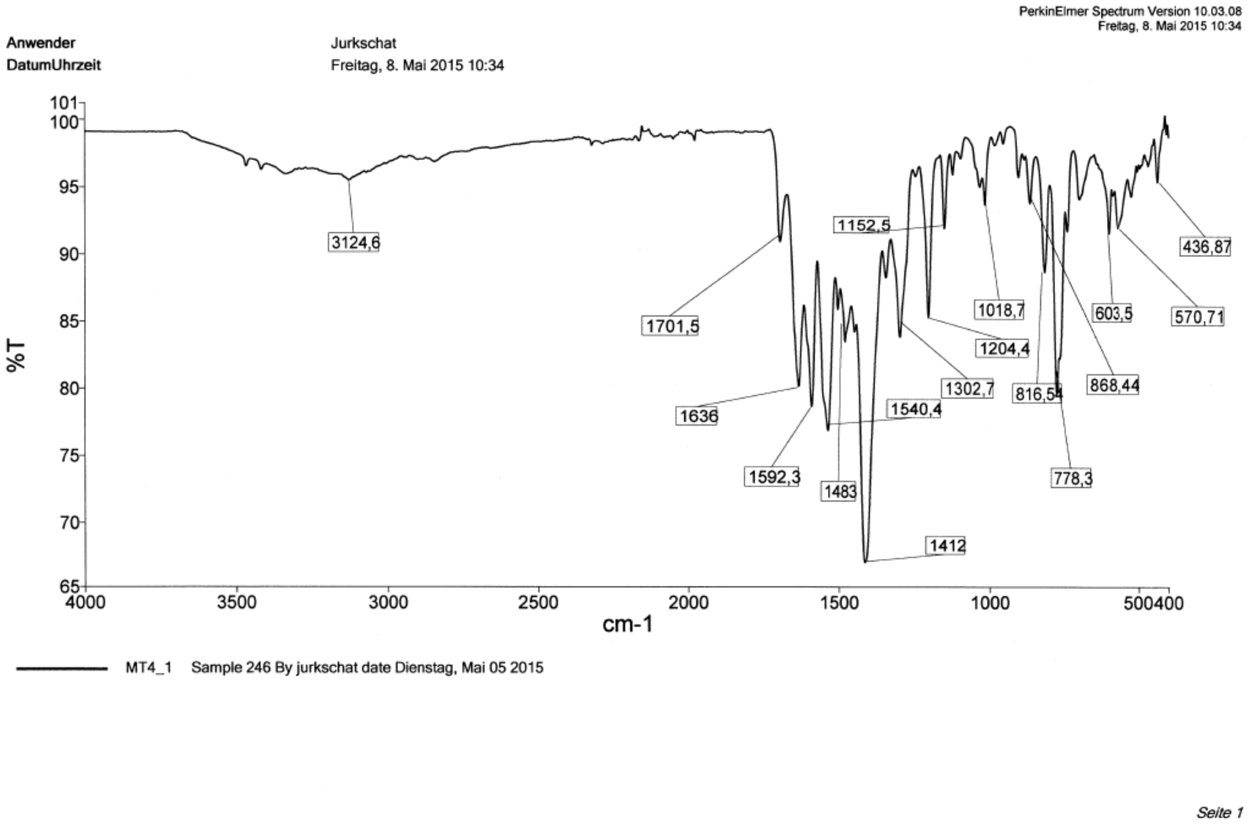

Figure S8: Infrared Spectrum of Gd(III)MT

This report was created by ACD/NMR Processor Academic Edition. For more information go to www.acdlabs.com/nmrproc/

04.05.2015 15:46:00

Acquisition Time (sec)	2.7329	Comment	z PROTON DMSO u quest 29			Date	29 Mar 2015 06:24:16		
Date Stamp	29 Mar 2015 06:24:16			File Name	C:\Users\Admin\Desktop\Nigeria\ACJ-ASW-MT\10\fid			Frequency (MHz)	300.13
Nucleus	1H	Number of Transients	16	Origin	spect	Original Points Count	16384	Owner	guest
Points Count	16384	Pulse Sequence	zg30	Receiver Gain	4096.00	SW(cyclical) (Hz)	5995.20	Solvent	DMSO-d6
Spectrum Offset (Hz)	1941.0437	Spectrum Type	STANDARD	Sweep Width (Hz)	5994.84	Temperature (degree C)	21.160		

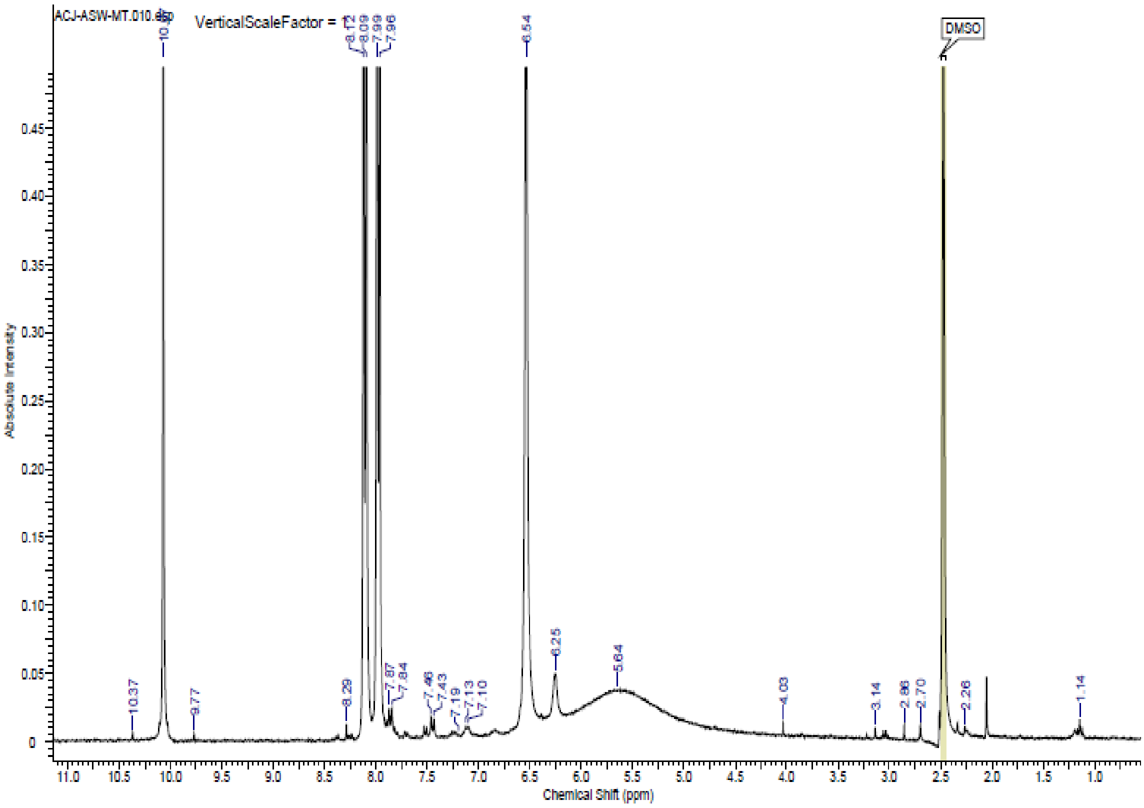

Figure S9: 1H NMR Spectrum of MT

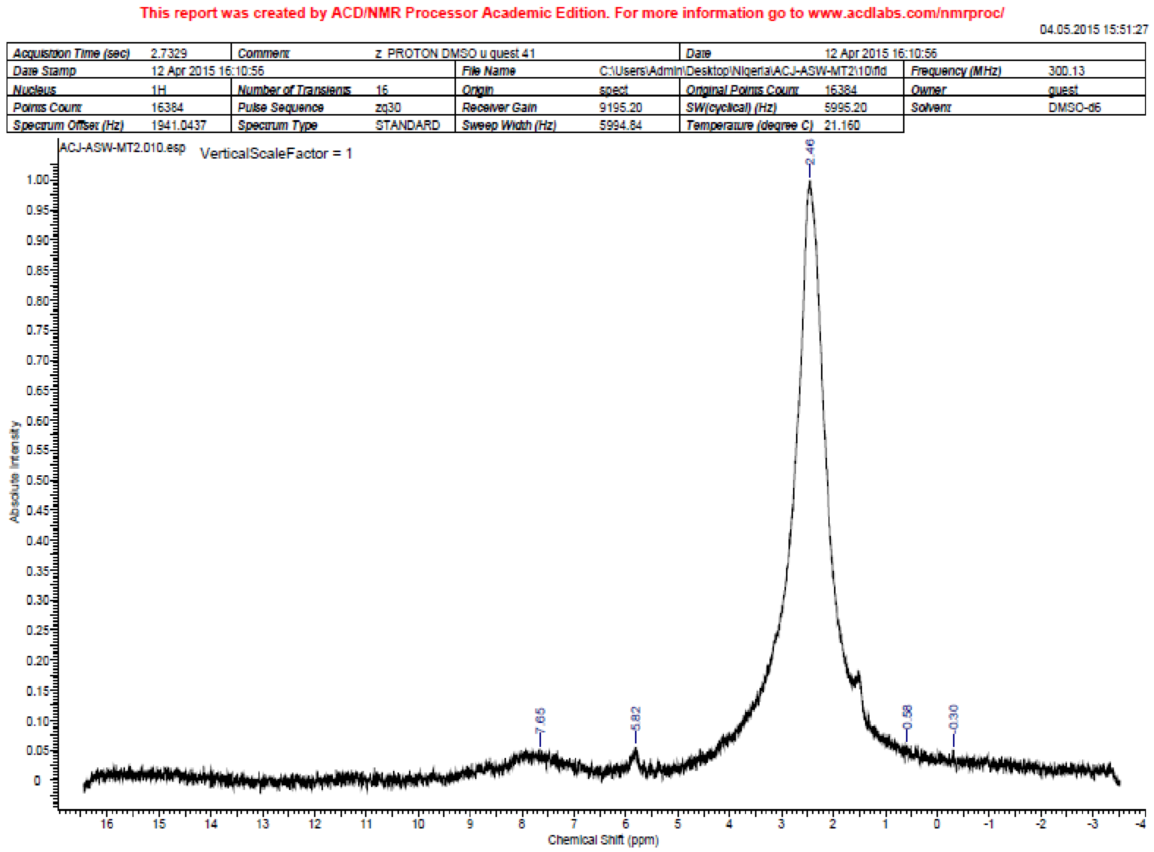

Figure S10: 1H NMR Spectrum of Dy(III)MT

This report was created by ACD/NMR Processor Academic Edition. For more information go to www.acdlabs.com/nmrproc/

04.05.2015 15:48:38

Acquisition Time (sec)	1.3763	Comment	z C13 CPD DMSO u quest 29			Date	29 Mar 2015 06:56:16		
Date Stamp	29 Mar 2015 06:56:16			File Name	C:\Users\Admin\Desktop\Nigeria\ACJ-ASW-MT\11\fid			Frequency (MHz)	75.47
Nucleus	13C	Number of Transients	640	Origin	spect	Original Points Count	32768	Owner	guest
Points Count	32768	Pulse Sequence	zgpg30	Receiver Gain	1625.50	SW(cyclical) (Hz)	23809.52	Solvent	DMSO-d6
Spectrum Offset (Hz)	9400.1836	Spectrum Type	STANDARD	Sweep Width (Hz)	23808.80	Temperature (degree C)	21.160		

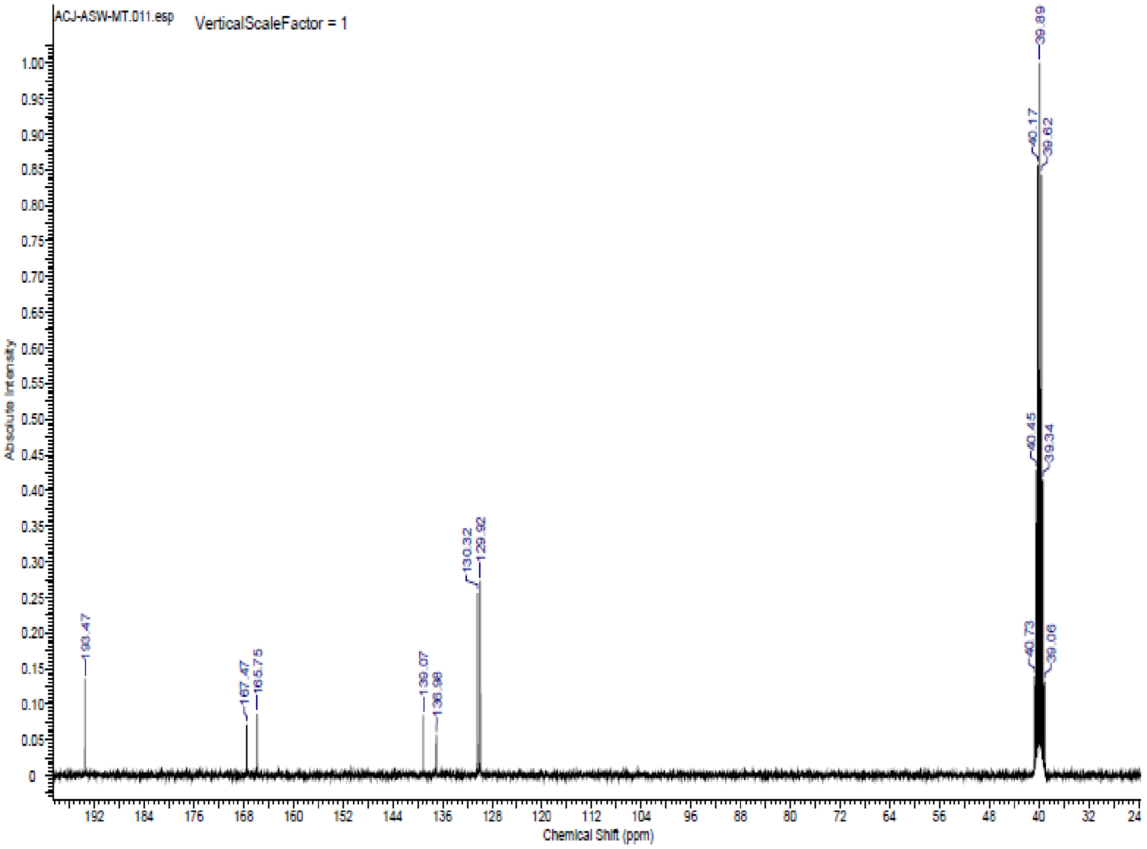

Figure S11: 13C NMR Spectrum of MT

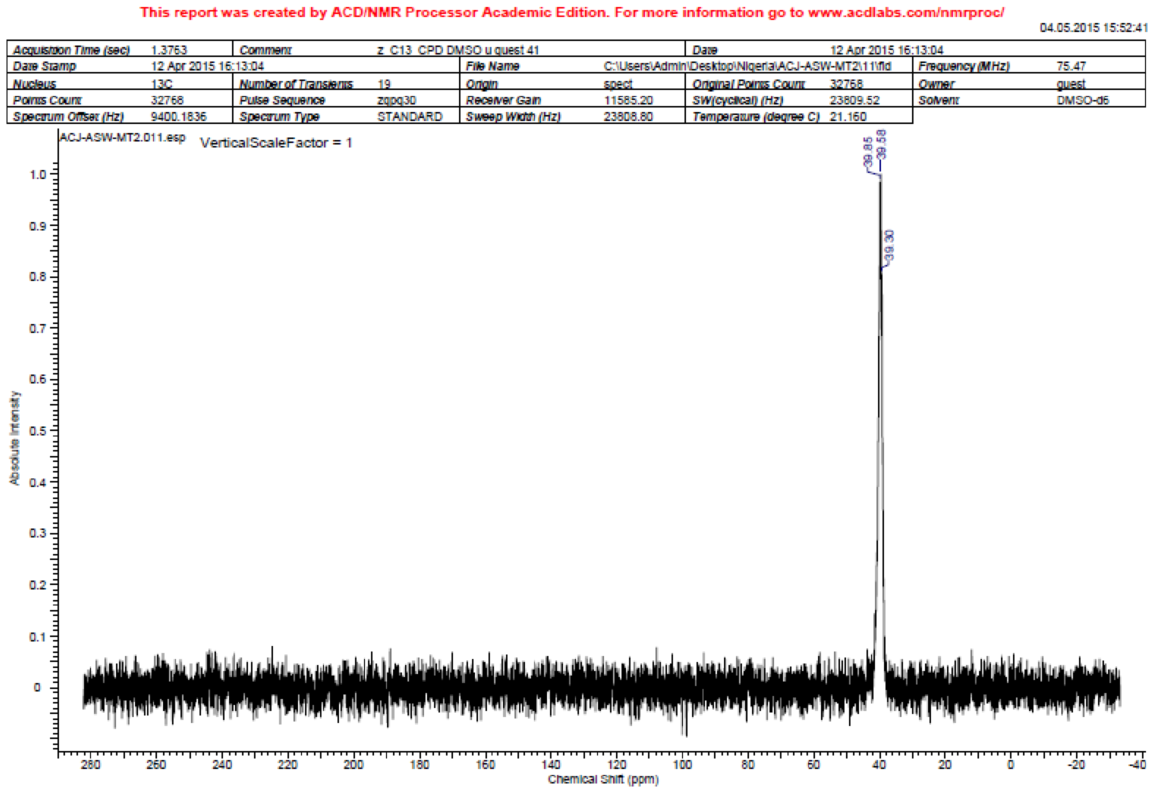

Figure S12: 13 C NMR Spectrum of Dy(III)MT

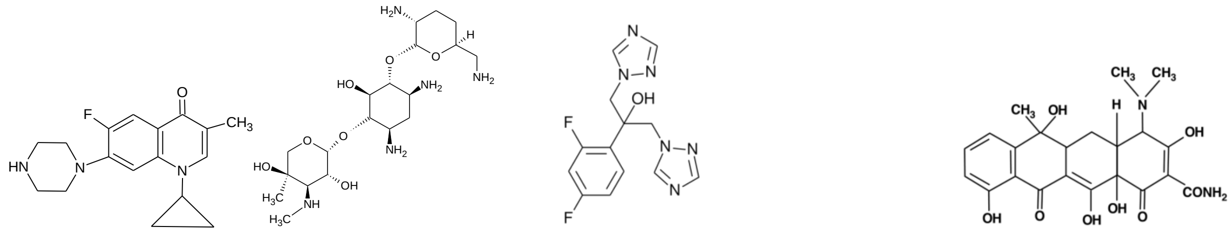

Ciprofloxacin Gentamicin Fluconazole Tetracycline

Figure S13: Structures of the drugs used as standard.

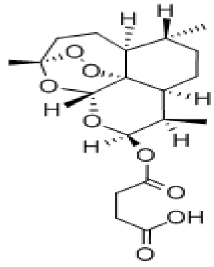

Figure S14: Structure of Artesunate.

Table S1: Inhibition Zone Diameter (IZD in mm) of the Controls

Conc. (µg/mL)	Code	*B.c* (ATCC 14579)	*S.a* (ATCC 6538P)	*P.a* (ATCC 9027)	*E.c* (ATCC 6749)	*C.a*	*A.n*

**12.5**	T1	11	13	20	14	15	20
**6.25**	T2	7	9	16	8	9	15
**3.125**	T3	3	5	11	3	4	14
**12.5**	F1	6	8	5	8	20	21
**6.25**	F2	3	5	3	5	15	17
**3.125**	F3	-	-	-	2	12	10
**12.5**	CP1	17	20	16	18	15	11
**6.25**	CP2	11	14	12	14	11	5
**3.125**	CP3	5	8	3	9	4	-
**12.5**	G1	19	12	20	14	16	21
**6.25**	G2	12	5	11	11	13	14
**3.125**	G3	5	2	8	2	9	10
**12.5**	DMSO	0	0	0	0	0	0
**6.25**	DMSO	0	0	0	0	0	0
**3.125**	DMSO	0	0	0	0	0	0

Legend: T = Tetracycline, F = Fluconazole, CP = Ciprofloxacin, G = Gentamycin.

Table S2: Determination of Acute Toxicity (LD_{50})

Samples	Animal mark	Wt(g) of mouse	Wt of sample (mg)	Vol of solvent (mL)	Conc. (mg/mL)	Time of death	Dose (mg/kg)
MT	Head	21.89	21.89	0.22	100	-	1000
	Tail	27.25	43.60	0.44	100	-	1600
	Trunk	26.38	76.50	0.38	200	-	2900
	R.Hind	25.53	127.65	0.64	200	-	5000
**Distilled water**	HEAD	21.02			100		5ml/kg

Table S3: Anti-plasmodic Effect of Samples on Albino mice

Sample/ Grp	% para-sitemia	Wt1	Wt2	PCV (%)1	PCV (%)2	Hb (g/dl)1	Hb (g/dl)2	Dose (mg/kg)
**MT**								
**A1**	12	29.49	32.18	53	49	16.5	15.5	25
**A2**	11	29.58	29.84	60	53	17.4	16.4	,,
**A3**	09	21.55	21.09	65	63	17.7	17.4	,,
**A4**	02	27.44	27.36	62	54	17.5	16.7	50
**A5**	07	24.01	25.22	67	62	17.8	17.4	,,
**A6**	06	20.55	25.16	64	58	17.6	16.7	,,
**-ve control(distilled water)**								
**B1**	29	29.05	27.01	63	59	17.20	16.50	5ml/kg
**B2**	38	25.07	20.33	67	43	17.50	14.00	,,
**B3**	27	25.81	25.27	64	51	17.40	15.10	,,
**+ve control(Artesunate)**								
**C4**	03	23.28	20.52	50	42	15.60	13.80	5mg/kg
**C5**	05	20.34	19.90	59	53	16.60	16.30	,,
**C6**	04	23.21	20.75	42	38	13.70	13.20	,,
